# Impact of UK Weather on Autonomous Vehicle Radar, LiDAR and Camera Vision Systems

**DOI:** 10.3390/s26175649

**Published:** 2026-09-05

**Authors:** Jessica Smith, Richard Dudley, David Jones, David Cheadle, Imran Mohamed, Fengping Li, Daniel Bownds, Hsun Yang, Joel Rapley, Andre Burgess, Mira Naftaly, Mayokun Aikomo, Jeremy Price, Nawal Husnoo, Stephan Havemann, Matthew Fry, Martin Osborne, James McGregor, Alan Vance

**Affiliations:** 1 National Physical Laboratory, Teddington, Middlesex TW11 0LW, UK; richard.dudley@npl.co.uk (R.D.); imran.mohamed@npl.co.uk (I.M.); fengping.li@npl.co.uk (F.L.); daniel.bownds@npl.co.uk (D.B.); hsun.yang@npl.co.uk (H.Y.); joel.rapley@npl.co.uk (J.R.); andre.burgess@npl.co.uk (A.B.); mira.naftaly@npl.co.uk (M.N.);; 2 Met Office, Exeter, Devon EX1 3PB, UK; dave.jones@metoffice.gov.uk (D.J.); nawal.husnoo@metoffice.gov.uk (N.H.); stephan.havemann@metoffice.gov.uk (S.H.); matthew.fry@metoffice.gov.uk (M.F.); martin.osborne@metoffice.gov.uk (M.O.); james.mcgregor@metoffice.gov.uk (J.M.); alan.vance@metoffice.gov.uk (A.V.)

**Keywords:** sensor performance, weather degradation, Autonomous Vehicle (AV), LiDAR, radar, camera, infrared, outdoor

## Abstract

Measurements are presented from a purpose-built outdoor test facility for the assurance of situational awareness sensors for Autonomous Vehicles (AVs). The testbed included meteorological instrumentation for an accurate high-resolution picture of weather traversing the site. Static targets at ranges up to 250 m were observed simultaneously with multiple radar, LiDAR and camera systems exposed to the natural weather events over a two-year period. The aim of the testbed was to collect data over a prolonged period of time to ensure the maximum amount of variation in weather was encountered. Uniquely, the testbed treats the measurement of weather as an equal metrology challenge to that of measuring the CAV sensor response with the aim of understanding the extent to which it is possible to quantitatively correlate sensor performance degradation with weather parameters. In this paper case study examples of the testbed data for rainfall, fog and ‘ideal’ neutral conditions have been analysed. Correlations between sensor performance degradation and weather parameters were observed in several cases, including comparisons with rain rate and MOR (visibility). However, large uncertainties and complex interactions between weather-related performance reduction and secondary weather effects, like sensor window and target surface wetting, make an explicit determination of the severity at which a weather condition causes a sensor to become untrustworthy, problematic.

## 1. Introduction

Results from an outdoor testbed are presented to quantify the performance of radar, Light Detection and Ranging (LiDAR), and camera systems exposed to UK rain, fog, and solar extremes. The paper begins with a review of the existing literature on sensors used for situational awareness (SA) within Autonomous Vehicles (AVs), followed by a detailed description of the testbed design and associated meteorological infrastructure. Finally, selected datasets from all three sensor types demonstrate the range of performance observed under different weather conditions.

### 1.1. Situational Awareness (SA) Sensor Background Review

AVs are a rapidly expanding market and by 2030 82% of all new vehicles sold globally are expected to have some level of automation performing one or more Dynamic Driving Tasks (DDTs) [1]. The increasing presence of AVs offers the potential to improve on-road safety by reducing human error when performing most DDTs. There were 1.19 million road traffic fatalities worldwide in 2021, many of which were caused by human error [2]. Statistics cite drivers failing to pay attention as a contributing factor in 38% of all UK motor fatalities in 2020, and careless driving (18%) and loss of control (11%) as other main factors. Factors that were not attributable to human driving behaviour, such as adverse weather causing a slippery road surface and pedestrian error, were only cited in 15% of UK cases [3]. However, it should be noted that inclement weather has proven to have a demonstrable impact on human driving behaviour. Becker et al. posited that precipitation, sun glare and high wind speeds are all contributing factors to crashes involving human drivers [4]. AVs could play a significant role in mitigating the driving hazards caused by human and environmental factors [5]. However, to make better decisions than a human driver, AVs require a level of ‘situational awareness’ appropriate for the DDTs they are designed to undertake. This awareness comes through a combination of sensors, most commonly radar, LiDAR, camera, Global Navigation Satellite System (GNSS) and ultrasonic sensors. Discrepancies in the performance of these SA sensors in real adverse weather conditions undermine this situational awareness, leading to reduced safety and performance if not accounted for.

Before deployment of any sensor, it is good practice to quantify performance and variability in isolation as part of a calibration or assurance process. Once deployed and operating outside a controlled test environment, the performance of any sensor will be compromised by three main factors: environment/weather, target variations, and sensor state. Weather events such as rain, fog and snow are the most obvious environmental conditions that can affect sensor performance and will introduce scattering and attenuation losses for all sensor types to different degrees. This reduction in performance is often the result of complex interactions between the electromagnetic spectrum used by the sensors and the weather at its finest scales, e.g., individual raindrops. Direct sunlight may also impact sensor performance, in particular LiDAR and camera, when the sun appears close to targets in the sensor’s field of view. Furthermore, in real-world conditions more than one weather event may take place simultaneously, for example fog under sunny skies, which might accentuate camera degradation due to ambient light scattering along the path between the target and sensor.

Sensor state includes factors such as drift in the sensor performance over time, which can reduce the accuracy of its output. This includes effects such as incremental degradation and mechanical fatigue during the lifetime of a sensor. It is, therefore, important to quantify the expected level of reduction in sensor performance over realistic timescales for an operating vehicle, and factor this into sensor calibration models. The physical position of the sensor on the vehicle, which could leave it exposed to and partially blinded by precipitation or frost and other elements such as dirt and dust build-up over time, can also affect the sensor state [6]. Such issues can be somewhat mitigated by using small heating elements or the sensor’s inherent dissipative heat, hydrophobic coatings and in situ cleaning such as wiper blades, though this may lead to long-term surface wear. The physical damage a sensor may experience over its lifetime should also be considered.

Quantifying weather degradation of a sensor enables uncertainties to be placed into platform vision and decision-making algorithms; for example, if the range of a camera is reduced from 100 m to 20 m by heavy rain, an intelligent vehicle must comprehend that its vision has been reduced and by what degree, so it does not falsely infer that there are no obstacles present over 20 m. Furthermore, for DDTs that require object detection and recognition, perception algorithms need to be trained on datasets that include example objects such as cars, pedestrians and bikes as they appear under adverse weather conditions to perform safely in real-world environments.

### 1.2. Related Work on Radar Performance in Adverse Weather

Many outdoor studies have been performed in the past 50 years on the effect of weather such as rain, snow and hail on radar [7,8,9] but the majority of these took place prior to 2000 and focused on radar operating at lower frequencies (<30 GHz) compared to current AV deployments at 77 GHz. These studies provide a groundwork for understanding the mechanisms by which these adverse weather effects can interact with and degrade signal performance. This understanding has been used to inform simulations of radar behaviour in adverse weather conditions [10].

More recent work on radar performance in adverse weather has focused on the creation of radar datasets that contain data taken in a variety of weather conditions, such as RADIATE [11], K-radar [12] and the Oxford Radar RobotCar dataset [13], to train algorithms for perception tasks. Whilst these datasets include data taken under conditions such as rain, fog and snow, they do not provide accurate meteorological measurements of the weather event alongside the radar data captures. Without quantitative weather data for the weather events included in these datasets it is difficult to make a judgement on whether the dataset spans a sufficient range of severities. For example, a dataset could include several instances of data taken in rain with the most severe rain event captured having a rate of 50 mm/h, unknown to the dataset user as rain data was not measured during the capture. If the area in which the user’s AV is required to work regularly experiences rain events above 100 mm/h, their perception algorithms may be insufficiently trained to cope with operational domain requirements.

Several studies on radar performance for perception tasks have also been carried out in artificial adverse weather conditions including a study by Li et al. [14] on the detection statistics of radar in rainfall and with varying light levels and a study by Steinhauser et al. [15] on the detection of pedestrians in rain.

### 1.3. Related Work on LiDAR Performance in Adverse Weather

Laboratory studies have been performed on LiDAR sensors for AVs in artificially generated weather conditions including rain [16,17,18] and fog [16,17,19] as well as secondary weather conditions such as spray water from other vehicles after rain [20,21]. These environmental chambers provide idealised experimental conditions for assessing sensor performance when exposed to a range of rain droplet sizes and densities. However, the interaction lengths, spatial variation and microphysical detail of artificially generated weather often do not match that of real weather and the performance of some sensors, notably radar systems, may be impacted by the walls of the chamber. Furthermore, secondary effects of the weather such as the generation of mud or slush during rain and snow may not be considered in such experiments unless they are the core topic of the study such as Vargas et al.’s [20] study on the effect of spray water.

Outdoor measurement campaigns for LiDAR [22,23] have also been conducted in rain [23], fog [19,22,23] and snow [19,22,23]. Of these campaigns, the fog measurements of Kutila [22] and Linhoff [23] and the snow measurements of Linhoff [23] were performed in the presence of several weather sensors to give data on variables such as visibility [22,23], precipitation rate [23], ambient temperature [23] and wind velocity [23]. It should be noted that in both cases, the weather variables were measured by a single sensor positioned close to the sensors under test. Dynamic vehicle-mounted tests such as those performed by Tang et al. for LiDAR and camera sensors [24] offer the most realistic scenarios, but from a metrology and measurement accuracy perspective offer an even greater challenge.

Several datasets for LiDAR perception algorithms have also been recorded in various weather conditions. Examples include the LiDAR Benchmark Reference (LIBRE) dataset which contains data from 10 different LiDAR sensors captured in a weather chamber where the sensors were exposed to fog, rain and strong light [25]. It was noted by Carballo et al. that the rain data captured was particularly inaccurate as the LiDAR captured the showers from the sprinklers within the environment chamber as vertical pillars. Other datasets, such as the A*3D which provides LiDAR and RGB Camera data taken from driving routes across Singapore, contain data taken under real weather conditions including sunny, cloudy and rainy [26]. However, the adverse weather data recorded is not exhaustive and includes only the weather that Pham et al. encountered along their planned driving routes.

### 1.4. Related Work on Camera Performance in Adverse Weather

Studies on camera performance in adverse weather have been undertaken within environment chambers such as the studies by Hasirlioglu et al. [27,28] who performed a variety of experiments with cameras in indoor rain simulations and the study by Velazquez et al. [29] that measured the performance of cameras, in this case thermal infrared cameras, in artificial fog.

Studies have been performed in real-world conditions such as the study by Tang et al. [24] that looked at camera performance as well as LiDAR in both rainy and sunny conditions with static and moving AVs.

Datasets, including labelled camera images, have been created with a significant focus on collecting data under real-world adverse weather conditions. An example of this is the Detection in Adverse Weather Nature (DAWN) dataset, which collected data in weather conditions including fog, rain, snow and sandstorms [30]. Once again, though this dataset contains a variety of weather conditions, no quantitative analysis of the weather variables is given. This dataset has been used by others to train their perception algorithms to perform object detection and recognition tasks under these challenging conditions. A study by Ashraf et al. used the DAWN dataset to test the performance of the You Only Live Once x (YOLOx) algorithm [31].

It is clear from the previous studies performed on sensor perception in adverse weather that the emphasis has either been on understanding the effect of a single type of weather on sensor performance under highly reproducible conditions, such as those studies performed within weather simulators, or collecting a variety of training data under real weather conditions with little reproducibility or weather metrology. The few studies that have been performed on sensor performance in outdoor environments under real weather conditions have been typically conducted on only one or two sensor types in locations that experience a large amount of extreme weather. The weather metrology performed within these studies is typically restricted to a few sensors positioned in one location.

### 1.5. Quantitative Description of Weather Conditions

Recent progress has been made in the description of some weather elements in the Operational Design Domain (ODD), the set of conditions within which an Autonomous Vehicle is designed to function. For example, ISO 34503:2023 (Road Vehicles—Test scenarios for automated driving systems—Specification for operational design domain) [32] refers to the need to specify the time and space intervals over which rainfall rate applies and points to the potential application of Meteorological Optical Range (MOR) measurements as a useful descriptor of fog, provided any loss of visibility can be unambiguously attributed to fog alone. ASAM OpenODD [33] specifies a modelling approach and exchange formats that enable the representation of ISO 34503-based (and other) taxonomies to be used in a practical sense. BSI PAS 1883:2025 [34] provides additional guidance on the unambiguous description of rainfall and wind parameters. Work is still required in optimising the representation of some of the ODD weather elements (notably solid precipitation) and, critically, in the appropriate use of these weather parameters when evaluating sensor response because meteorological standards for weather measurement were not designed with this application in mind. In particular, the uncertainties that arise from the natural variability of some parameters over time and space scales are not generally well handled in the context of sensor characterisation. This is problematic because, for example, rainfall may impact sensor performance due to a path-integrated attenuation impact and/or the instantaneous highly localised effect of sensor/target wetting, yet it is typically measured as a time-integrated point measurement located some distance away from the sensor. A rigorous handling of these uncertainties is essential if experimental data from physical testbeds are to be used in the sensor assurance process.

### 1.6. Motivation of This Study

In order for testbed assessments of sensor performance to be quantitatively useful for safety assurance, it is essential to consider their limitations. This includes understanding: the degree to which *all* of the environmental factors that might affect the sensor response have been either measured or estimated and any remaining uncertainties quantified; the limitations of conventional meteorological variables as predictors of sensor performance; and, finally, the limitations of the meteorological instruments themselves. This is true whether using physical weather simulators to produce controllable (and repeatable) measurements or using outdoor testbeds to capture the naturally occurring variations observed in nature. Our observation is that existing studies tend to under-recognise these limitations and that meteorological measurements made by single weather sensors are taken too much at face value. This can result in either overconfidence in the sensor characterisation or in finding it difficult to explain when the sensor performance appears to correlate poorly with measured weather.

The purpose of the testbed, built by the National Physical Laboratory and the Met Office, and presented within this paper, was to observe the impact of ‘real’ weather on sensor performance whilst also understanding the challenges around the ‘meteorological metrology’. To the best of the authors’ knowledge, the testbed and measurement campaign represents the most comprehensive sensor performance study so far conducted in terms of the meteorological metrology with major weather variables being studied by multiple sensors positioned at multiple locations over the testbed site. This allows for a thorough study of the local variation and uncertainty in weather variables as well as in sensor performance, something that is not well explored by previous real or simulated studies.

Furthermore, this paper makes an important distinction between the weather observations made by individual pieces of meteorological instrumentation (the *measurements*) and the testbed-wide parameters that can be derived from them (the *measurands*). This is important because it allows us to consider the limitations of the weather measurements and acknowledge measurement error, including whether we have adequately sampled time and space variability across the testbed. It also allows us to combine multiple measurements to produce an improved estimate of the weather parameter and its uncertainty, especially where the accuracy of the measurement may itself be weather-dependent. The final parameters and their uncertainties are then unambiguously defined, which is critical if they are to be compared to results from other testbeds or used in safety assurance. In short, the paper treats the measurement of weather as an equal metrology challenge to that of measuring the CAV sensor response.

The ultimate aim of this study is to understand to what extent an outdoor testbed, with both sensor and meteorological variables measured and their uncertainties quantified, can explain and quantify the behaviour of situational awareness sensors in different types of weather. From this information, we hope to draw conclusions as to what extent an explicit determination of a weather parameter value over which a sensor’s output can no longer be considered trustworthy is possible as an approach to setting ODD limits. In this paper, we consider these questions through the analysis of radar, LiDAR and camera performance under three weather conditions: ‘flat’ or neutral weather, rain and fog. For brevity we consider the sensor performance on three case study days: a flat weather day, a day with two rain events and a day with a fog event.

This paper is separated into six sections. Section 2 details the design of the sensor testbed including: a description of the testbeds operation, an introduction to the web portal on which the testbed data has been made publicly available, information on the types of sensors deployed including the targets and analysis methods used with them and the different RF and visible wavelength attenuation reference measurements taken. Section 3 details the meteorological measurements performed on the testbed including the types of meteorological measurement equipment deployed and their positions on the site, what parameters this equipment was used to measure and discussion on the treatment of some frequently used weather parameters: rain rate, radar attenuation in rain and MOR (visibility) where some more intricate or unique methods of calculation have been employed. Section 4 presents sensor performance data for each of the sensor types (radar, LiDAR and camera) on each of the case study days (flat/neutral weather, rain and fog). The correlations between weather parameters and sensor performance are presented and discussed. Section 5 contains discussion on what the results of these measurements mean for the explicit determination of ODD parameters and Section 6 is the conclusion.

## 2. Testbed Design: Sensors and Meteorological Monitoring

**Figure 1:**
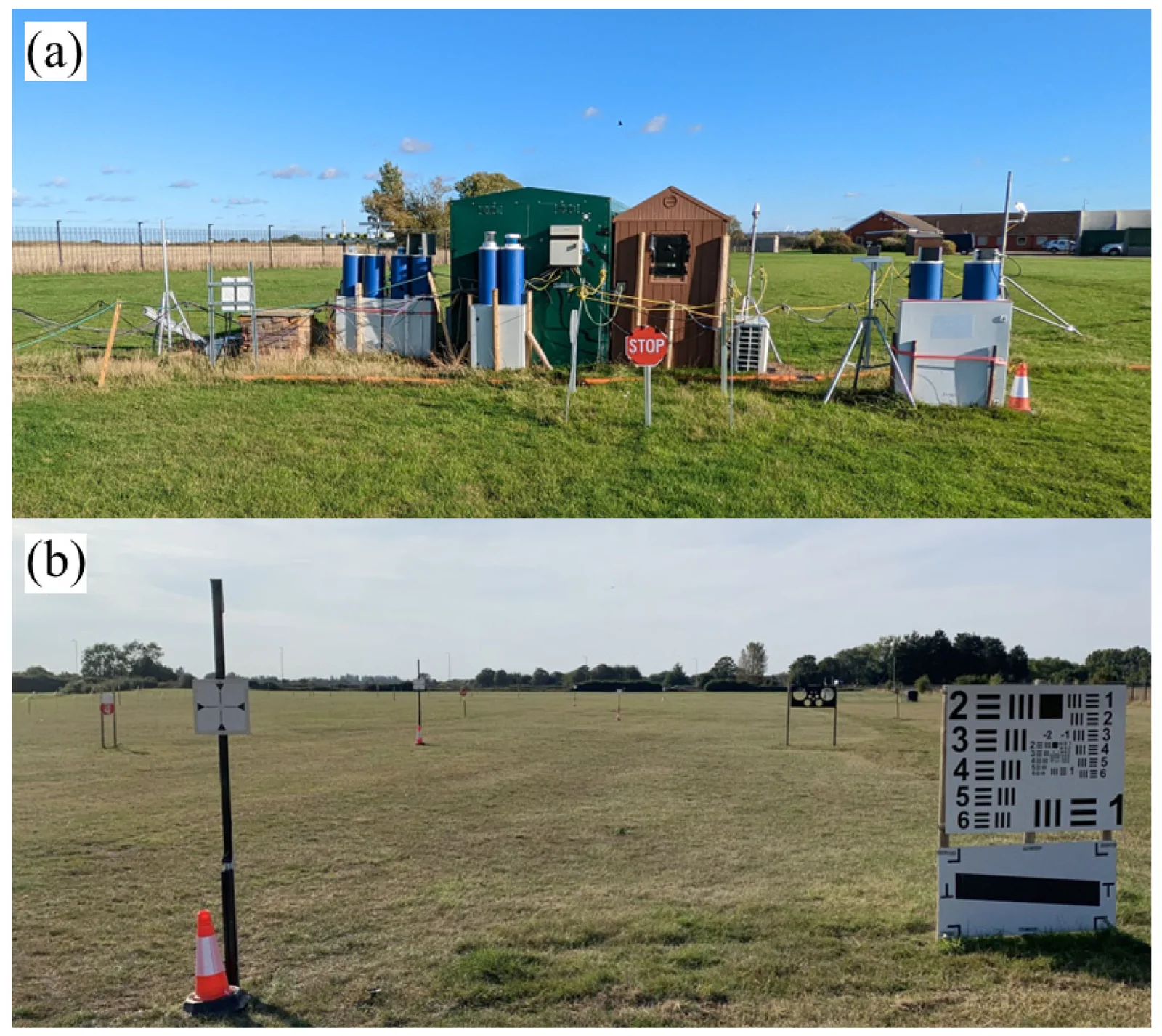
Testbed: (**a**) Testbed control building and installed cameras, LiDAR and radar. (**b**) View down the testbed as seen from sensors in (**a**); targets to left are LiDAR/radar with a camera resolution chart on the right.

The measurement testbed was part of a UK programme to assure SA sensors by combining the skills of the UK’s National Physical Laboratory and the Met Office [35]. The testbed combined an array of static targets deployed over a 250 m open space, observed simultaneously by multiple LiDAR, radar and camera systems, Figure 1. The downrange targets enabled calibration of sensors’ range, resolution at range, target reflectivity, and spatial variability. The accurate position of targets was verified through a site survey with cm accuracy, supported by aerial imagery. In parallel two atmospheric loss systems at optical and microwave frequencies measured path loss over the target space to isolate contributing factors of sensor window and target wetting to recorded data.

The UK’s Met Office provided the testbed site, which already had meteorological apparatus deployed with data records stretching back to 1998, and for this project that was complemented with the addition of an array of research-quality measurement systems and a small network of typical operational measurements in order to capture time and space variability across the site.

The testbed operated twenty-four hours a day between January 2022 and March 2024, with any gaps being due to sensor restarts, failures, or servicing. To demonstrate the breadth of data taken during the testbed operation, the section ‘Sensor Performance in Adverse Weather’ presents sensor performance results for case study days with ‘flat’ weather (overcast but rain- and fog-free), heavy rainfall and fog. Only a single instance of each weather event is presented here for each sensor type; however, more data from the testbed has been made available through an online data portal. In total the Cardington site experienced 206 h of significantly reduced visibility (less than 1000 m) due to mist or fog (occurring over 102 separate days) and 1062 h of rainfall (occurring over 495 days and totalling 1428 mm).

Time synchronisation between all sensors, SA and meteorological, was provided by two GPS time servers with an accuracy of milliseconds, far faster than the needed one-second accuracy of the testbed.

**Figure 2:**
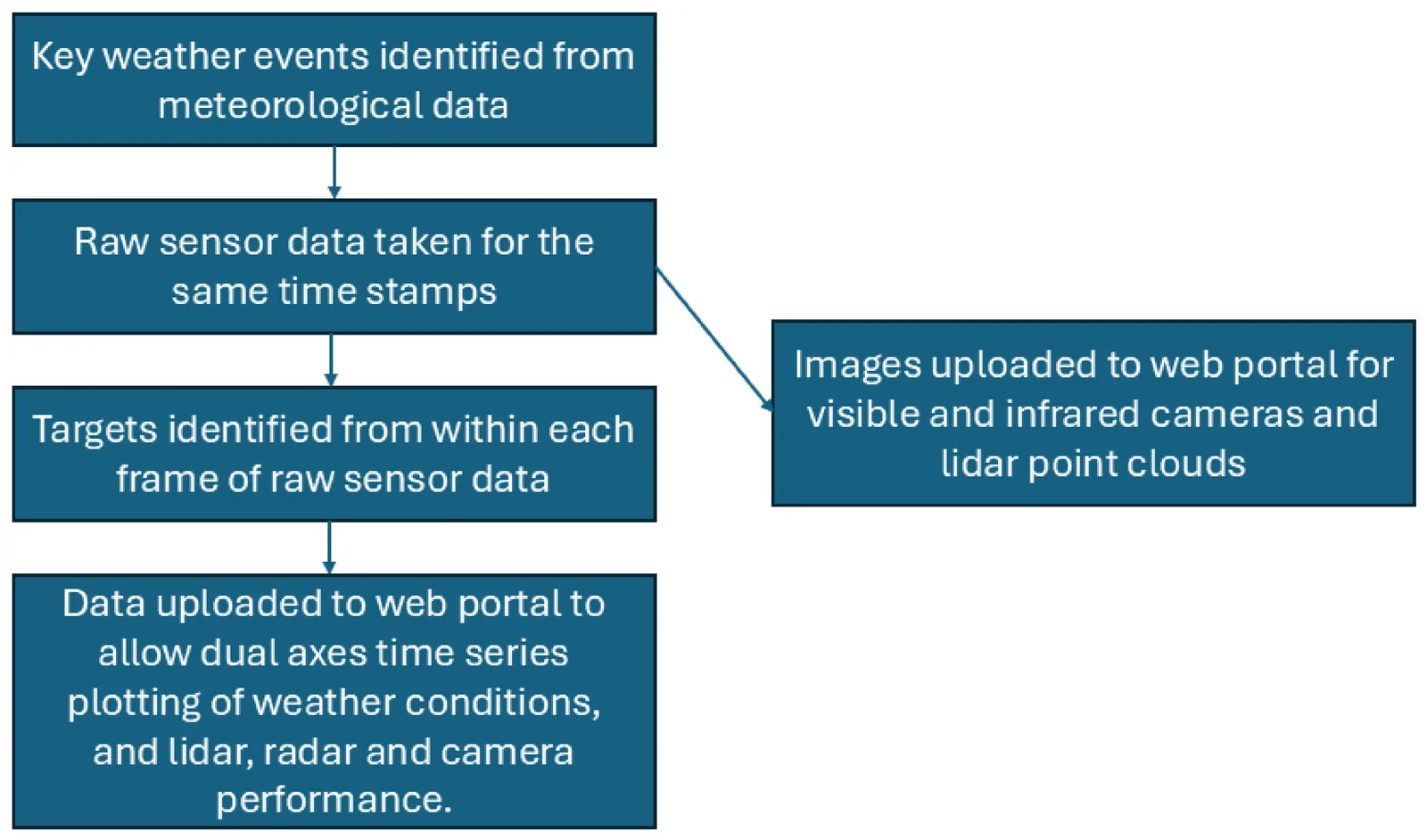
Flow diagram of how data is added to the Sensor Assurance Framework data portal.

Raw, curated and summary datasets are available to the community for those working on virtual test environment models, sensor uncertainty and sensor noise analysis. Curated data has been published at https://saf.npl.co.uk/ (accessed on 28 August 2026) and enables browsing to selected measurement dates, producing plots of meteorological and sensor data for inspection and comparison. Figure 2 shows a flow diagram outlining how data has been selected and processed for display on the web portal.

### 2.1. Deployed SA Sensors and Analysis Methods

Both 2D and 3D radars were deployed on the testbed. Here results are presented for a dual-beam 2D device operating at 76–77 GHz. The long-range beam has a 250 m range and ±9° field of view and the short-range beam extends to 70 m with a field of view of ±60°. Performance was measured by analysing the returned radar signal from multiple corner reflector targets positioned downrange on the testbed. The returned signal from individual reflectors was isolated and variations in signal, RCS and position measured over time.

**Figure 3:**
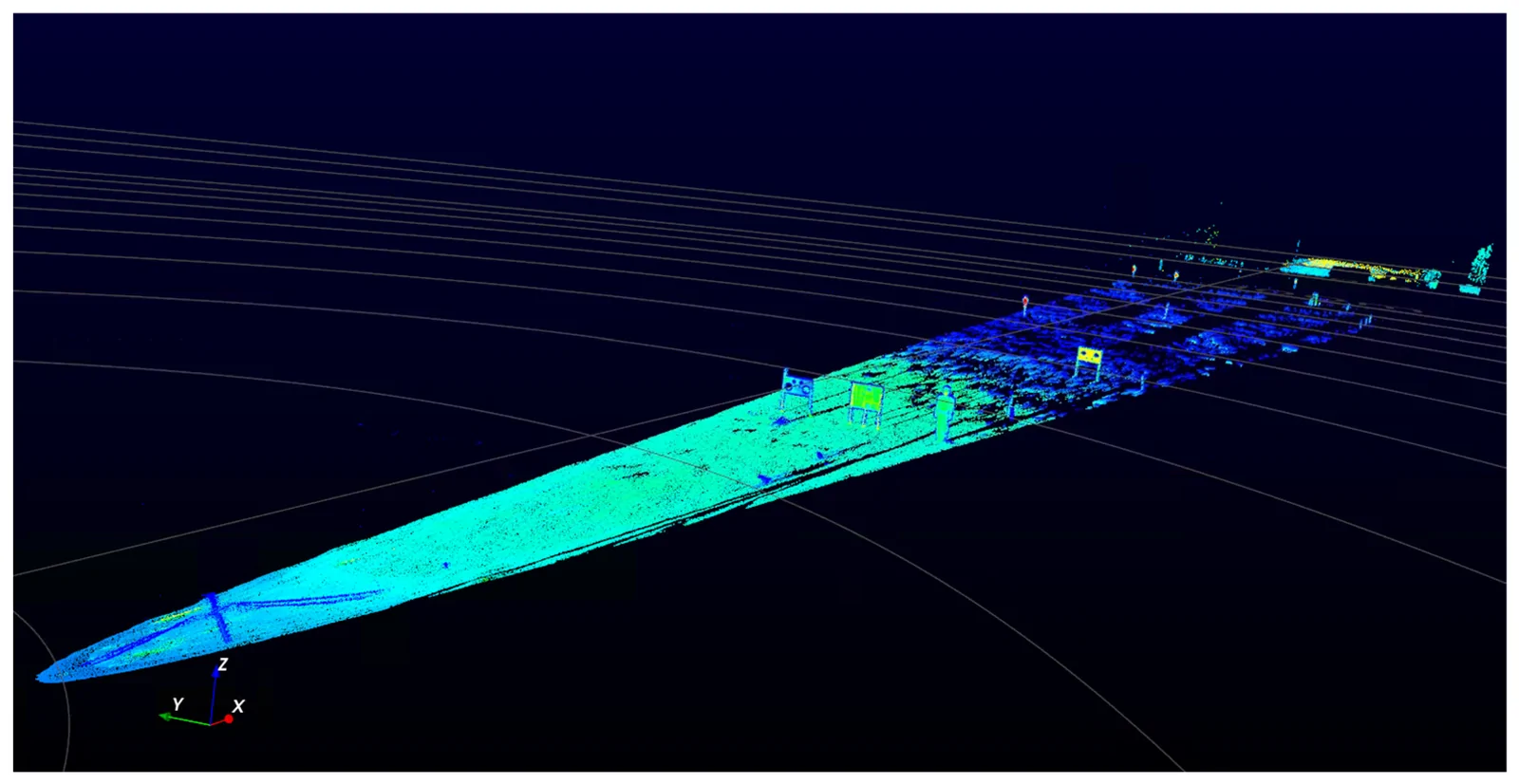
LiDAR image of the test site with visible objects highlighted out to 250 m. Green to light blue points indicate high reflectivity, dark blue points indicate low reflectivity.

Multiple LiDAR sensors covering commercial wavelengths, scanning mechanisms and ranges were deployed on the testbed. For this paper, data are presented for a single 360° mechanical scanning LiDAR operating at a wavelength of 903 nm with a range of 220 m. This range is based upon the distance at which a 10% reflectivity target can be detected with at least three returns in the point cloud. LiDAR performance was measured by analysing the reflected signal from fixed, high-reflectivity targets placed at multiple distances along the testbed. An example of a measured point cloud is shown in Figure 3 along with the axis definitions. On each target point-cloud data is isolated by applying a 3D bounding box around its known centre. The reflectivity of a target is calculated using a distance weighted average from its centre, with the number of returned points providing a measure on confidence and visibility.

As with other sensor types, multiple cameras were deployed but only one device is presented here, a 5.4 MP (2880 × 1860) 3 µm pixel and a 25 mm focal length lens, creating a 20° × 13° (horizontal × vertical) field of view. Multiple targets were installed and, in this paper, we focus on analysis of a high-contrast slanted edge target to obtain spatial frequency response (SFR). The slanted edge feature is taken from the larger version of the SFR target defined in ISO 12233:2000 [36]. SFR is a metric that quantifies the amount of contrast information within a scene that is transferred into the final image, and how it varies with the size of the features within a scene.

MATLAB code sfrmat5 [37] (running on MATLAB 2024) was used to calculate SFR for the camera images where the target was visible. Images with too low a contrast due to, e.g., low-light levels, or where the target was obscured due to, e.g., water droplets on the window, were omitted from the analysis. SFR data is presented for the camera’s operation under ‘flat’ weather, rain and fog conditions. To evaluate how the camera system’s SFR is affected by these conditions, the behaviour of four values derived from the SFR and defined by the IEEE 2020–2024 standard [38] are studied:

- SFR10: The spatial frequency at which the SFR value equals 10%, the Rayleigh criterion for limiting resolution.
- SFR50: The spatial frequency at which the SFR curve equals 50%, a commonly used metric derived from SFR plots.
- SFR at 0.25 Nyq: The value of SFR at one-quarter of the Nyquist frequency or 0.125 cycles/pixel.
- SFR at 0.5 Nyq: The value of SFR at one-half of the Nyquist frequency or 0.25 cycles/pixel.

### 2.2. Reference Path Loss Systems

Alongside the automotive SA sensor systems there were also two separate reference systems deployed at the testbed site to directly measure the optical and radio frequency path loss using metrology-grade equipment. These systems were included to provide reference measurements under different weather conditions that the SA sensors’ performance could be compared against.

#### 2.2.1. Optical Path Loss System

**Figure 4:**
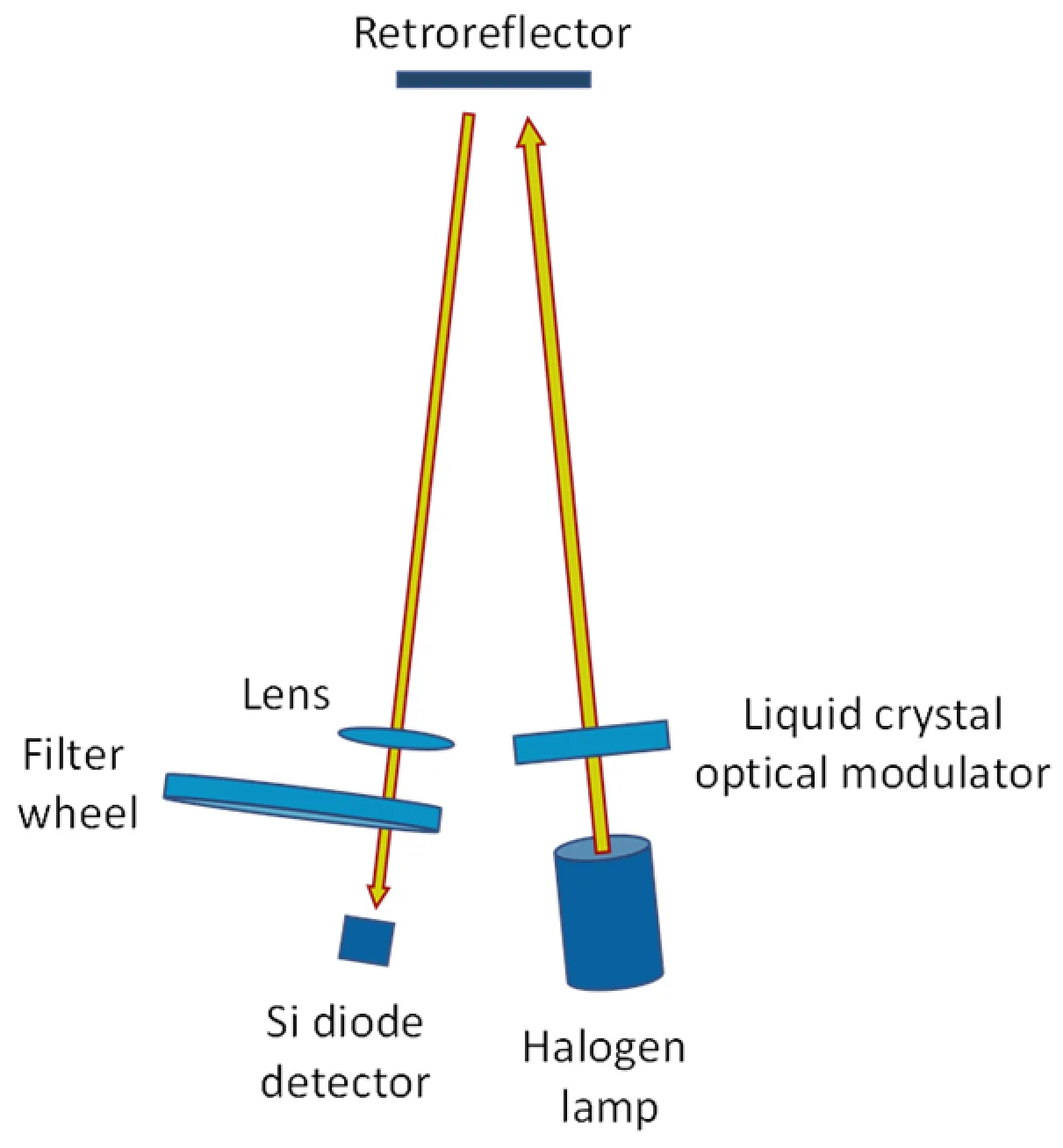
Schematic representation of the optical path loss system.

The optical path loss system (OPLS) measures the atmospheric losses in the visible-to-near-IR spectrum over a fixed 100 m range, Figure 4. The OPLS employs a wide-spectrum light source in the form of a quartz tungsten halogen lamp (Thorlabs QTH10, Thorlabs, Bergkirchen, Germany) with a liquid crystal amplitude modulator. The beam is directed to a retroreflector located at 47.5 m downrange; the reflected beam is focused by a lens onto a silicon photodetector (Thorlabs PDA100A2, Thorlabs, Bergkirchen, Germany). A filter wheel in front of the detector enables wavelength selection and intensity calibration. The wheel contains 6 filters: four narrow-band interference filters centred at 400 nm, 520 nm, 650 nm, and 800 nm; and two neutral density filters at Optical Density (OD) = 1 and OD = 2. A lock-in amplifier (LIA) is used to remove noise due to fluctuations in the background illumination, and the captured signal is logged by a PC with a time stamp for each data point. The optical assembly and signal-processing electronic instruments are enclosed in a weatherproof and air-conditioned housing with an optical window providing access to the signal beams.

The system was designed to be sufficiently sensitive to quantify transmission loss in low-visibility conditions; it cannot measure atmospheric attenuation in high-visibility conditions, which is of the order of 0.02 dB/km in the visible region. The maximum signal level recorded by the instrument therefore represents high-visibility low-attenuation atmospheric conditions. In conditions of reduced visibility, attenuation in dB/m can be calculated from the recorded signal.

#### 2.2.2. Radio Frequency Path Loss System

The second reference system was the radio frequency path loss system (RFPLS). This system operates with a centre frequency of 77 GHz to match one of the main frequencies used by automotive radar, the other being 24 GHz. The deployed RFPLS is a two-part system consisting of separate transmitter and receiver stations. The transmitter has a radio frequency (RF) signal generator and an external frequency multiplier module, and the receiver consists of an RF power sensor. Both the transmitter and receiver are equipped with 25 dBi standard gain horn antennas and are weather-protected. The system measures the change in RF path loss over 150 m making measurements of attenuation at 76.0, 76.5, and 77.0 GHz. The system collected measurement data every 30 s by transmitting a continuous wave tone and measuring the received signal power.

**Figure 5:**
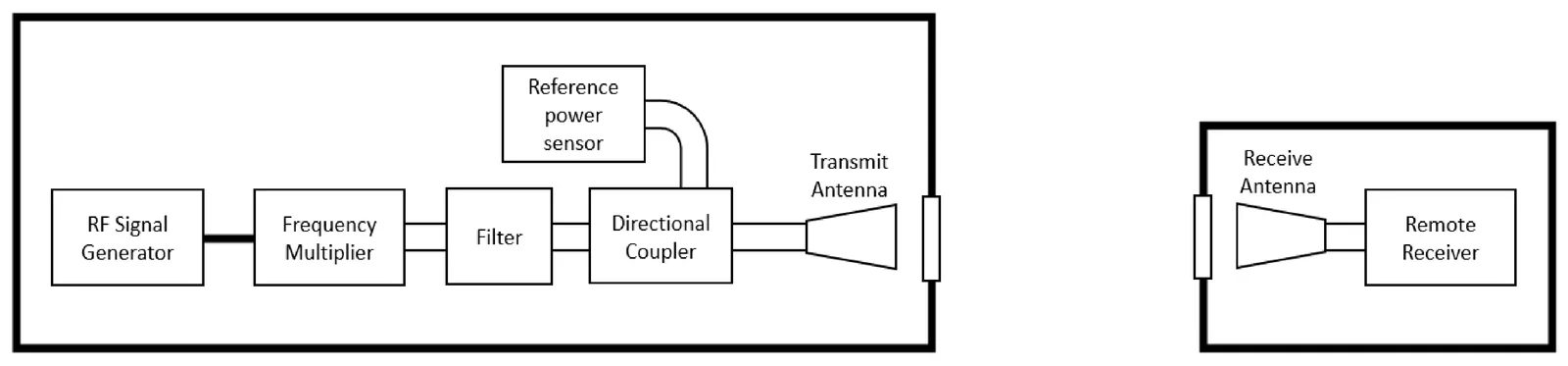
Schematic representation of the radio frequency path loss system, shown in the final configuration, in which the filter, directional coupler and reference power sensor were added.

The RFPLS was operational between March and December 2022 and rebuilt and re-deployed for a second set of measurements taken during 2024. The re-deployed version of the system was updated to include a directional coupler and a power sensor on the transmitter to allow the transmitter power to be measured; a schematic of this version of the system is presented in Figure 5.

## 3. Meteorological Measurements and Data

The purpose of the meteorological measurements at the testbed was to firstly understand *what* measurements are useful, and secondly to derive meaningful meteorological parameters from these measurements for comparison with the SA sensor output. The following principles guided the choice and placement of the meteorological instrumentation:

1. All of the known meteorological sources of significant sensor degradation were measured by some means (including aerosol, fog, precipitation, thermodynamic variables and also short- and long-wave radiation).
2. The microphysical detail (such as droplet size spectra) of atmospheric hydrometeors was sampled in detail at least in one location.
3. The spatial and temporal variability of key weather parameters across the testbed were characterised by repeating selected measurements in multiple locations. This variability was represented in uncertainty estimates for key weather parameters, where such uncertainty may be consequential in characterising the sensor response.
4. Given the eventual application of these measurements to real-world deployment, the weather measurements included both research-quality and more conventional operational instrumentation.

**Figure 6:**
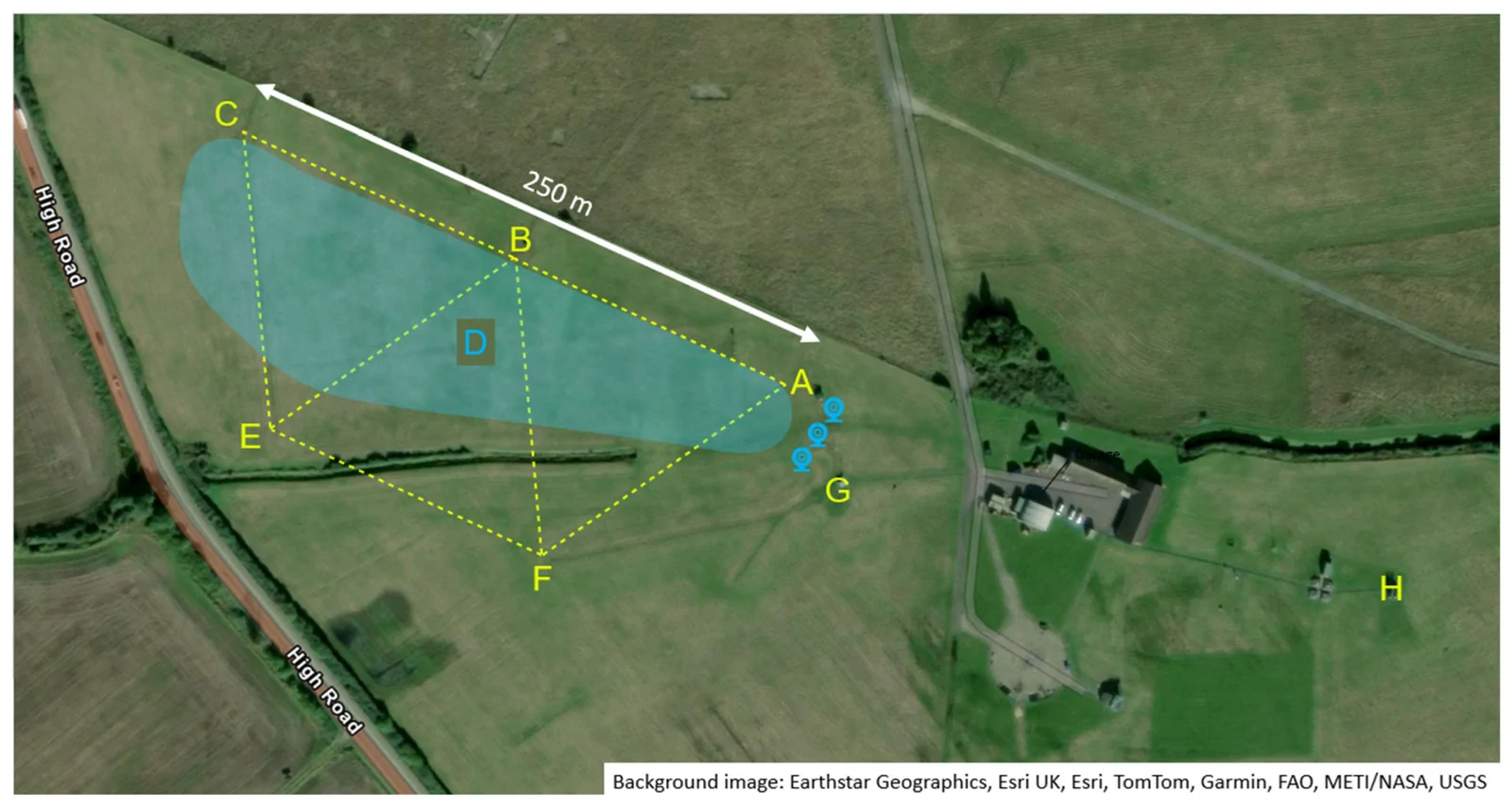
Testbed site schematic. Yellow letters indicate the locations of meteorological instrumentation. Blue symbols represent the indicative location of the SA sensors that observed targets distributed within the shaded area D.

The locations of meteorological measurement sites are indicated in the testbed site layout shown in Figure 6. SA perception sensors were all located close to Point A and observed the previously described SA sensor reference targets contained within shaded area D in the figure. The sensor and targets were largely bounded by the trapezium ACEF, where the length of AC was 250 m. Point B represents the midpoint of the line AC and Points A, B, C, E and F were arranged on the vertices of equilateral triangles of side 125 m. In this paper, when we refer to ‘testbed’ values of meteorological parameters, we mean values that are representative of the area contained within and immediately adjacent to the trapezium. Point G represents the approximate measurement location for the measurement of the thermodynamic variables and Point H the location of Cardington’s radiation reference site.

### 3.1. Testbed Instrumentation

Table 1 summarises all of the meteorological instrumentation deployed, their locations and their function on the testbed.

### 3.2. Curated Testbed Parameter and Uncertainty Values

A selection of parameters relevant to the specific weather conditions rain and fog have been used within this paper and their definitions are provided in Table 2.

For parameters such as rain rate and MOR, the variation across the site may be both significant meteorologically *and* consequential to the performance of an SA sensor. For example, fog patchiness during its formation or dissipation phases may result in very different MOR values in one part of the testbed compared to another.

As well as the spatial aspects of the meteorological measurement, it is also necessary to consider the integration time over which the meteorological parameters are evaluated. This requires a compromise that considers the following factors:

1. Some weather measurements (e.g., disdrometers) require a finite time to create a statistically meaningful measurement.
2. Averaging over too long a period may lose important temporal detail.
3. For parameters such as rainfall that vary spatially, a longer integration window effectively makes the measurement more representative of the path-integrated value that is relevant to understanding attenuation between SA sensor and target.
4. There is benefit in using sampling times that are likely to be used in the operational meteorological networks as it is these that will be used to monitor the weather (known as Current Operational Domain, COD) when the SA is deployed.

There is no ‘correct’ time period, merely an informed pragmatic choice, and it is critical to clearly define this choice in the description of the derived weather parameter. All the weather parameters for the testbed represent one-minute integration periods and should be interpreted as the expected value of that parameter if it were to be measured at a random point within the trapezium ACEF (this choice is a compromise that resolves the time variability of key weather parameters but with a reasonable integration period to sample, for example, raindrop size distributions, and is commonly used as a sampling period in conventional meteorological observing systems). The associated uncertainty, given by error bars in the following plots, reflects the impact of both variability within that area and the inherent error characteristics of the source measurements themselves, with the larger of these two factors dominating the error. These 1 min parameters and uncertainties are then the basis of all the plots shown, regardless of the actual positions of AV sensors and targets within the testbed.

### 3.3. Rainfall and Attenuation Processing Approach

To fully characterise the response of SA sensors to the weather, the meteorological measurements themselves must be understood, including their error characteristics. Rainfall presents a challenge because of its fractal-like variability in both time and space and the diversity of measurement approaches, including bulk (collecting rain volume in rain gauge); particle-based (e.g., disdrometers); and remote (such as an operational rainfall radar with a pixel area of the order of km^2^). The SA sensors themselves respond to several different properties of the rainfall, such as the along-path attenuation (experienced as a series of wavelength-dependent interactions with individual raindrops); multipath effects; the wetting of sensor surfaces and targets; or noise due to backscatter from the droplets.

The strength of disdrometers is that, by capturing the drop size distribution (DSD) itself, it is possible to gain insight into rainfall rate and sensor-specific attenuation at the same time. However, the finite sampling area of the devices coupled with a finite sampling time interval means that the measured DSD must be considered just one possible sample from an overarching DSD at the location of the disdrometer (the sampling area of devices of this kind decreases for larger droplets, as they are more likely to only partially occlude the detection beam). Likewise, the measurements from the network of five measurements are samples from the spatially varying DSD field over the testbed. In uniform moderate rainfall (such as dynamic or ‘frontal’ rain), we might expect these two sources of uncertainty to be at their smallest; in showery (convective) rainfall, where precipitation is more localised and often falls as fewer larger droplets, the uncertainties will increase. If attempts are made to infer data-driven sensor impact models from testbed data, where the greater impacts might be at these higher rainfall intensities, it is imperative to understand these uncertainties.

It is beyond the scope of this paper to fully describe the quality control and error estimation process; however, the following steps were taken to extract rainfall DSDs from the disdrometer data.

1. Quality control (following [39]) was applied to the 1 min disdrometer droplet velocity/size counts. The filtered values were seen to give good agreement when compared with the accumulated rainfall measured by the tipping bucket rain gauge at Point B.
2. The uncertainties arising from the finite sampling area and time were estimated by treating the number of counts per droplet size bin as the mean value of a Poisson distribution, which could then be resampled to simulate sets of statistically consistent bin counts. By repeating this exercise across all size bins 200 times, 200 plausible DSDs for a single disdrometer measurement were produced, from which 200 samples of rainfall rate and associated attenuation (at all four SA sensor wavebands) were calculated. Conceptually this may be considered to be the same as the range of measurements that 200 identical disdrometers placed in close proximity to the physical measurement might measure.
3. This was repeated for all five disdrometers to create 1000 samples of testbed rainfall rate and attenuation. These 1000 samples approximate the spread of measurements that would be acquired if 1000 disdrometers were distributed randomly across the testbed.
4. The mean and standard deviation of these 1000 samples were then taken as the representative testbed value and uncertainty for the rain rate and attenuation coefficients.
5. This then becomes our unambiguously defined rainfall rate parameter, which is the expectation value and standard deviation of a 1 min disdrometer rainfall (and attenuation) measurement placed anywhere on the testbed.

For completeness we note that using the guidance of BSI PAS 1883:2025, our rainfall rate would be described as “Sampling Area” is [“Point”] AND “Time interval” is [60 s]. Currently the standards do not provide guidance on expressing the uncertainty component.

The above treatment is, of course, only a model but it produces the desirable behaviour of exhibiting larger uncertainties in intense, spatially inhomogeneous convective rain and much lower uncertainty in light or moderate dynamic rain. However, as with all error modelling it is susceptible to the assumptions used and error bars may be exaggerated when there are low counts at larger droplet sizes. We note here that the attenuation calculations assume spherical droplets. Also, the attenuation coefficients are derived from single-particle total extinction values (which include both scattering and absorption). In the case of the LiDAR and photopic wavebands, much of the scattering by the raindrop will be directed forwards and the attenuation coefficient may therefore overstate the energy lost from the beam. At 77 GHz, the attenuation coefficients can safely be interpreted more literally and so we restrict predictive plots to the radar only.

### 3.4. Meteorological Optical Range (MOR) Processing and Plotting Approach

The World Meteorological Organisation (WMO) defines the visibility parameter MOR as the length of path in the atmosphere required to reduce the luminous flux in a collimated beam from an incandescent lamp, at a colour temperature of 2700 K, to 5% of its original value. The luminous flux is evaluated by means of the photometric luminosity function, as defined by the Commission Internationale de l’Eclairage (CIE), which describes the average spectral sensitivity of human visual perception of brightness [40]. Almost all practical means of estimating MOR involve making localised measurements of physical quantities that can be converted to MOR by some means. Each has strengths and weaknesses, which are wholly dependent on the underlying cause of the visibility loss. In fog and ‘flat’ cases, this paper uses the MOR values measured by the CS125 visiometers as this represents the regime most suited to this kind of measurement. To highlight this choice, these measurements are denoted MOR_fog_ in the figures. In the rain case, the extinction coefficients derived from the disdrometer and FM120 drop size spectra are used to calculate MOR, and the resulting value is denoted MOR_rain_. Note that the term ‘MOR’ is used in the body of the text, without reference to the measurement method. This is consistent with the principle of making a distinction between measurement and measurand—the physical quantity is always MOR, regardless of the source.

When plotting sensor response in relation to MOR, a practical choice for the upper limit of MOR to resolve useful detail while covering a reasonable dynamic range is required. In this paper, the OPLS (1-way) path length was 100 m and the longest (2-way) LiDAR attenuation path was 98 m. For these configurations an MOR value greater than 5000 m corresponds to an intensity reduction of approximately 5% or less at optical wavelengths and was therefore chosen as the upper plotting limit for MOR.

### 3.5. Selected Weather Event Days

To demonstrate the variation in sensor performance under different weather conditions, sensor data is presented in this paper for three scenarios, a flat/stable day, rain and fog days. The flat data offers an ideal calibration day where cloud cover is high and dense, light levels vary little and there is no noticeable precipitation or condensing humidity. The flat day provides a baseline sensor performance and inherent uncertainty.

Flat weather days here include 5 March 2022 for radar and 13 September 2022 for LiDAR and camera. Both days were overcast but clear of weather with no significant rain or fog. Data for 5 March 2022 is presented between 6:37 am (sunrise) and 12:00 pm. During this day, a few isolated traces of precipitation were measured, all with a rain rate of <0.06 mm/h and MOR values above 20 km. Data for 13 September 2022 between 12:00 pm and 17:00 pm demonstrated overcast conditions with no rain and fog and MOR greater than 20 km.

Rain data is presented for 20 October 2022 for all sensors, a day with significant rainfall, including the heaviest rate the UK typically experiences. The highest rainfall occurred between 6:30 am and 8:00 am with a peak rate of 112 mm/hour at 6:14 am.

Fog formation at Cardington is primarily formed by overnight cooling in very still conditions. As such, there were only a few events where fog conditions, illumination levels and sensor availability were simultaneously suitable. In this paper, two case studies are presented: 18 March 2022 (for radar) and 26 August (for LiDAR and camera). The day of 18 March 2022 had a significant fog event between 1:00 am and 8:30 am. During 26 August 2022, fog occurred throughout the night and into the morning, dispersing at around 8:00 am. During this event, the visibility, as defined by the measured MOR_fog_ value, dropped as low as 49.0 m at 5:18 am. Camera and LiDAR data are presented from 5:00 am on this day.

## 4. Analysis of AV Sensor Performance in Adverse Weather

This section contains post-processed data for each SA sensor observing downrange targets during three different weather conditions: ‘flat’ weather, rain and fog. Data includes, where available, the two path loss systems, optical and RF/microwave.

### 4.1. RADAR

Radar returns from downrange targets encompass a single or few data points containing range, angle and power returned. Filtering of noise/clutter is achieved through a power threshold set point and two-dimensional bounding boxes at the target locations to isolate returns. The mean radar signal from each target is plotted over time with corresponding weather variations to identify correlations.

#### 4.1.1. RADAR: Flat Weather

**Figure 7:**
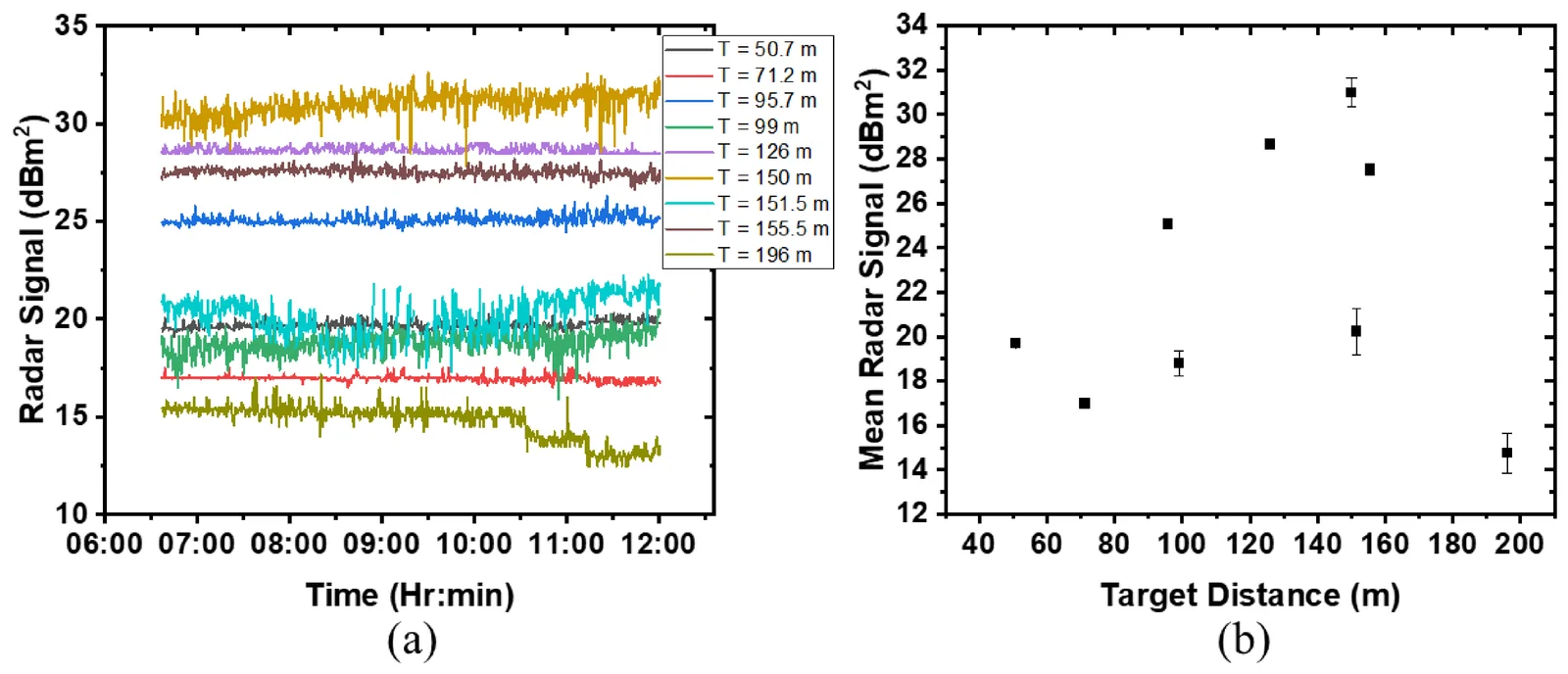
(**a**) Reflectivity from nine targets at ranges T from 6:30 am on 5 March 2022; (**b**) mean reflectivity over five-hour window for each target. Error bars are standard deviation of the reflectivity over the five-hour window in (**a**).

The returned signal from nine radar targets for a ‘flat’ weather scenario on the morning of 5 March 2022 is shown in Figure 7a and the mean values and range shown in Figure 7b. The nine radar targets were varied in design and include corner reflectors as well as small and large marine radar reflective targets. The radar signal varied most for targets furthest away, with 4 dBm^2^ for targets at 151 m and 196 m after 11:00 am, but below 1 dBm^2^ (5%) for most of the morning. As no significant weather event is present during this period and other targets at similar distances, such as 126 m and 150 m, do not appear to show such variation, it is unlikely to be weather-related and could be due to variation in the target structure or possibly secondary effects such as targets moving or flexing due to wind or temperature. The largest variation in reflectivity was 6.67 dBm^2^ for the 196 m target, after 11:00 am, with the smallest, and close to typical, variation for the 126 m target of 1 dBm^2^. These values represent the inherent error of the radar system when operating in the most stable conditions.

#### 4.1.2. RADAR: Rainfall

**Figure 8:**
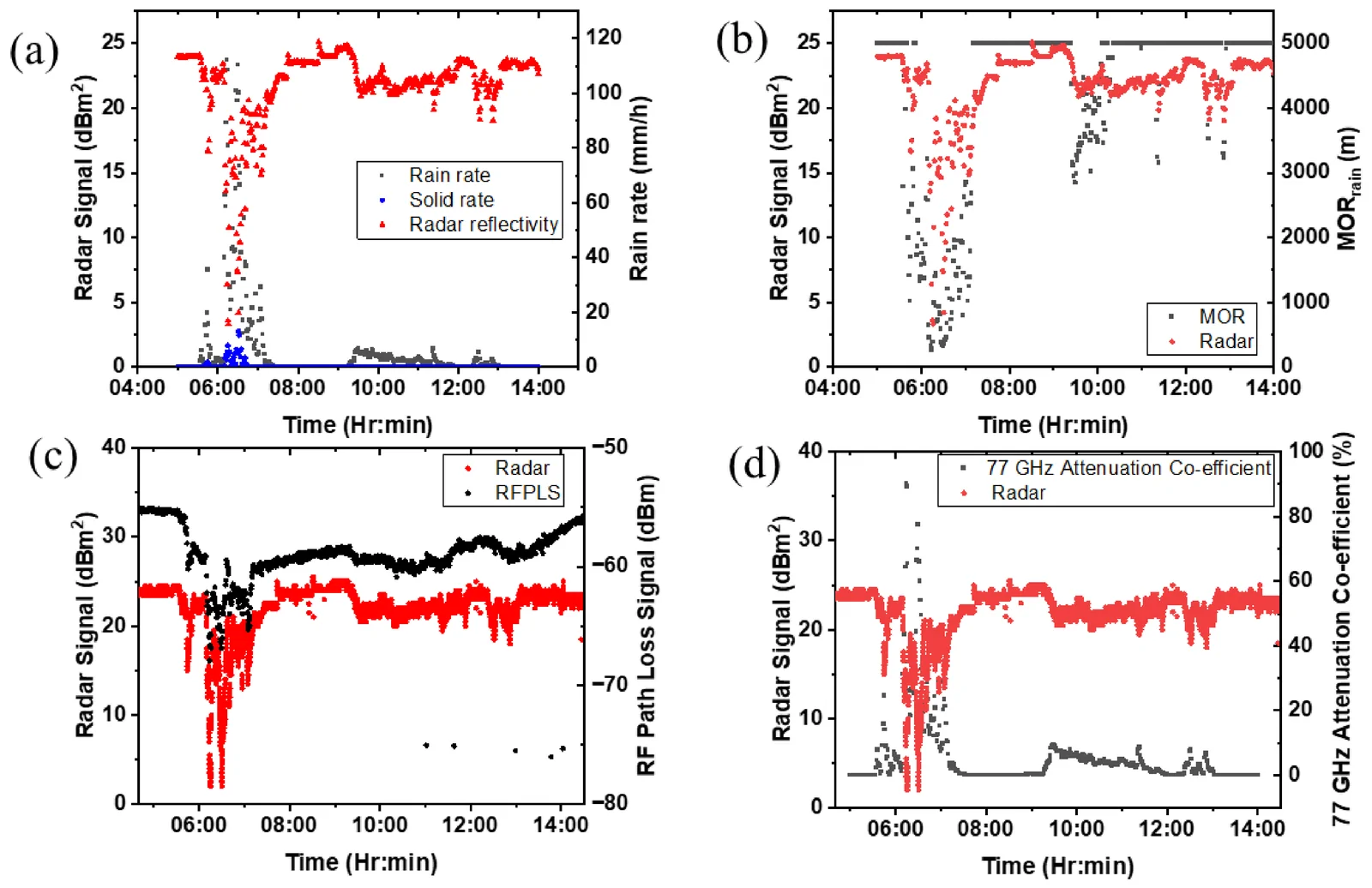
Radar signal strength vs. (**a**) rain rate, (**b**) MOR, (**c**) NPL RF path loss and (**d**) disdrometer 77 GHz calculated attenuation coefficient.

The returned radar signal for a target at 175 m range for 20 October 2022 is presented in Figure 8 with corresponding rain rate, MOR_rain_, NPL 77 GHz RF path loss system and disdrometer-inferred 77 GHz attenuation coefficient.

The data for 20 October 2022 is significant as it contains a period of the highest rainfall rate experienced at the testbed of over 100 mm/h at 6:30 am, corresponding to the largest radar return drop of 20 dB, almost obscuring the target. Note that during this event some hail was also present. Figure 8a also highlights a time lag in the recovery of the radar signal after the rain events. Comparing the radar signal and MOR, Figure 8b, there is good agreement; however, the drop in radar signal for the second rain event appears to be sustained over the course of the whole rain event whilst the MOR value dips at the beginning of the rain and when there is a small peak in the rain rate. Such a difference could be due to secondary effects, such as droplets on target surfaces and sensor windows, lingering after the rain. Figure 8c shows a comparison of the radar signal and the RF path loss signal which shows that the radar signal recovers more rapidly than the RF path loss signal after a rain event. Further discussion on this topic is given at the end of the section.

Figure 8d is a comparison of the radar signal and an attenuation coefficient at 77 GHz, calculated from raindrop size distributions measured on the testbed. This shows an increase in attenuation during rain events, with a much more significant increase during the heaviest rain period.

When looking at the correlations of the meteorological variables with the sensor data, it is important to consider the difference between correlation and causation. It can be seen from Figure 8b that the radar signal decreases during the periods of reduced MOR as the rainfall impacts both visible and microwave wavelengths. However, comparing the first and second rain event, it can be seen that the relationship between MOR, which is a measure of attenuation at visible wavelengths, and the radar signal changes. This is due to the strong wavelength and droplet size dependence of attenuation and demonstrates the limitations of some meteorological parameters to serve as predictors of sensor performance. Put simply, we would not expect to express the ODD of a radar sensor in terms of MOR.

**Figure 9:**
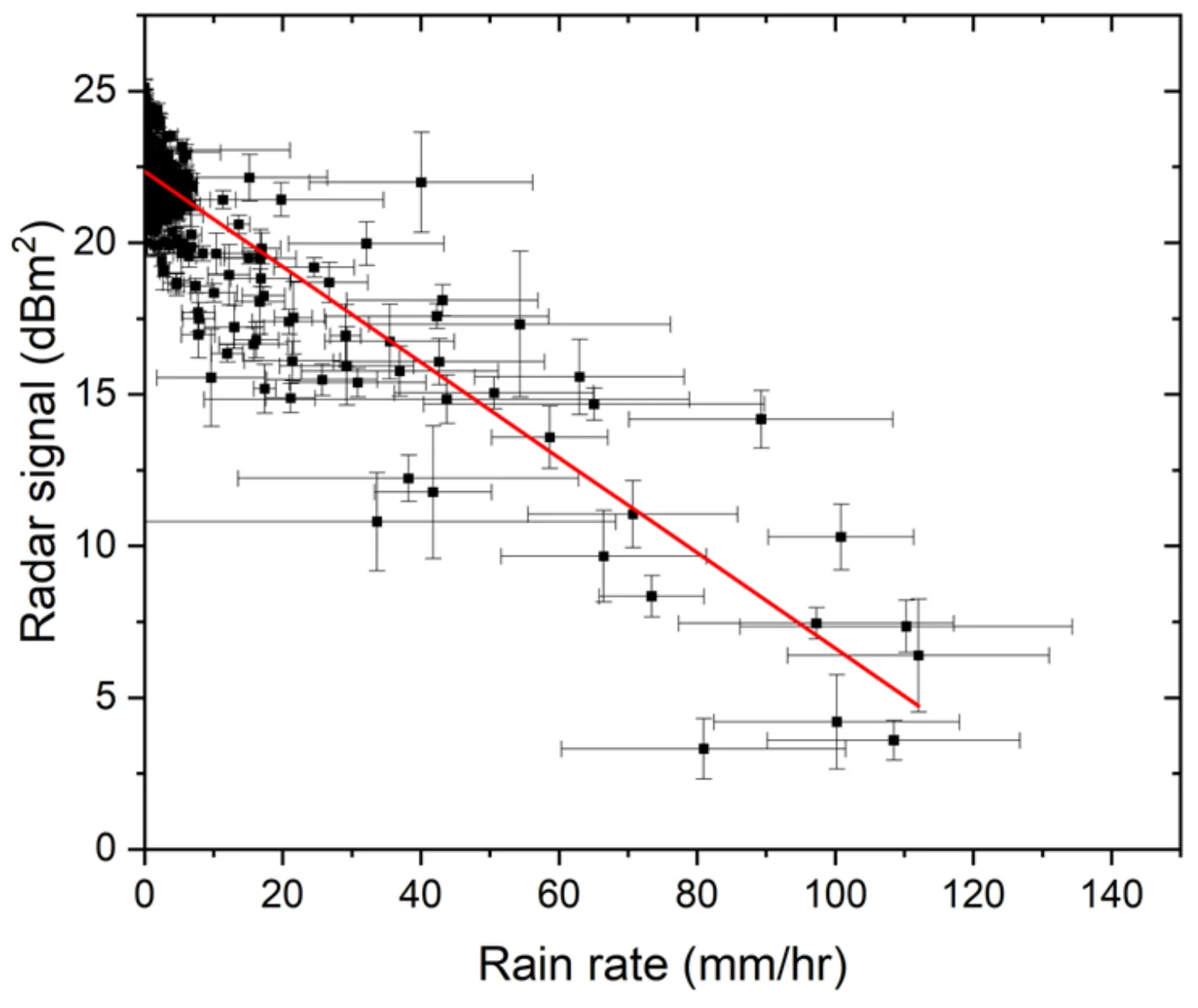
Radar signal returned from a reflective target as a function of rain rate during the 20 October 2022 rainfall. Each point represents one-minute mean and standard deviation of radar reflectivity and corresponding value of the 1 min testbed rainfall value. The red line shows a linear fit that has been applied to this data.

The rain-rate-versus-target-radar-signal plot, shown in Figure 9, shows a strong correlation between the reduction in radar signal and the rain rate. A linear fit was applied to this data (shown by the red line in the figure). The slope of this fit was calculated to be −0.157 ± 0.003 dBm^2^/(mm/h). While the correlation is strong, it is notable that there are points that lie some distance from the line, particularly for the highest rain rates. This is important because it suggests that such a linear relationship has some limitations if it is to form the basis of a data-driven simulation of radar performance in rain.

**Figure 10:**
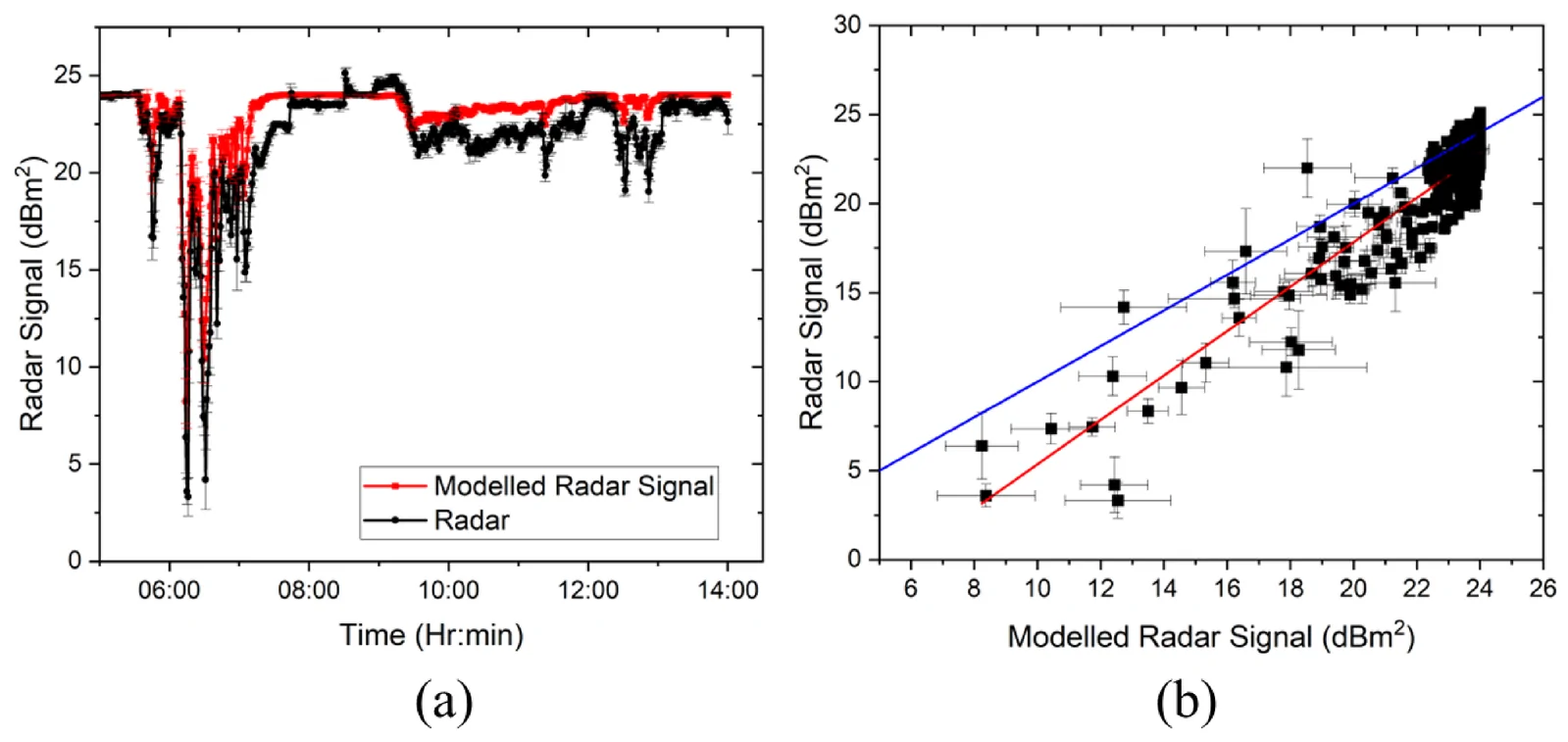
(**a**) The modelled radar signal strength in dBm^2^ based on the 77 GHz attenuation coefficient calculated from raindrop size distribution during the 20 October rain event compared to the measured radar signal strength over the same time period and (**b**) the modelled signal strength versus the measured signal strength. A guideline (blue) has been provided to show the line x = y which all points should lie along if the modelled data accurately explained the measured data. A linear fit of the data is shown in red.

**Figure 11:**
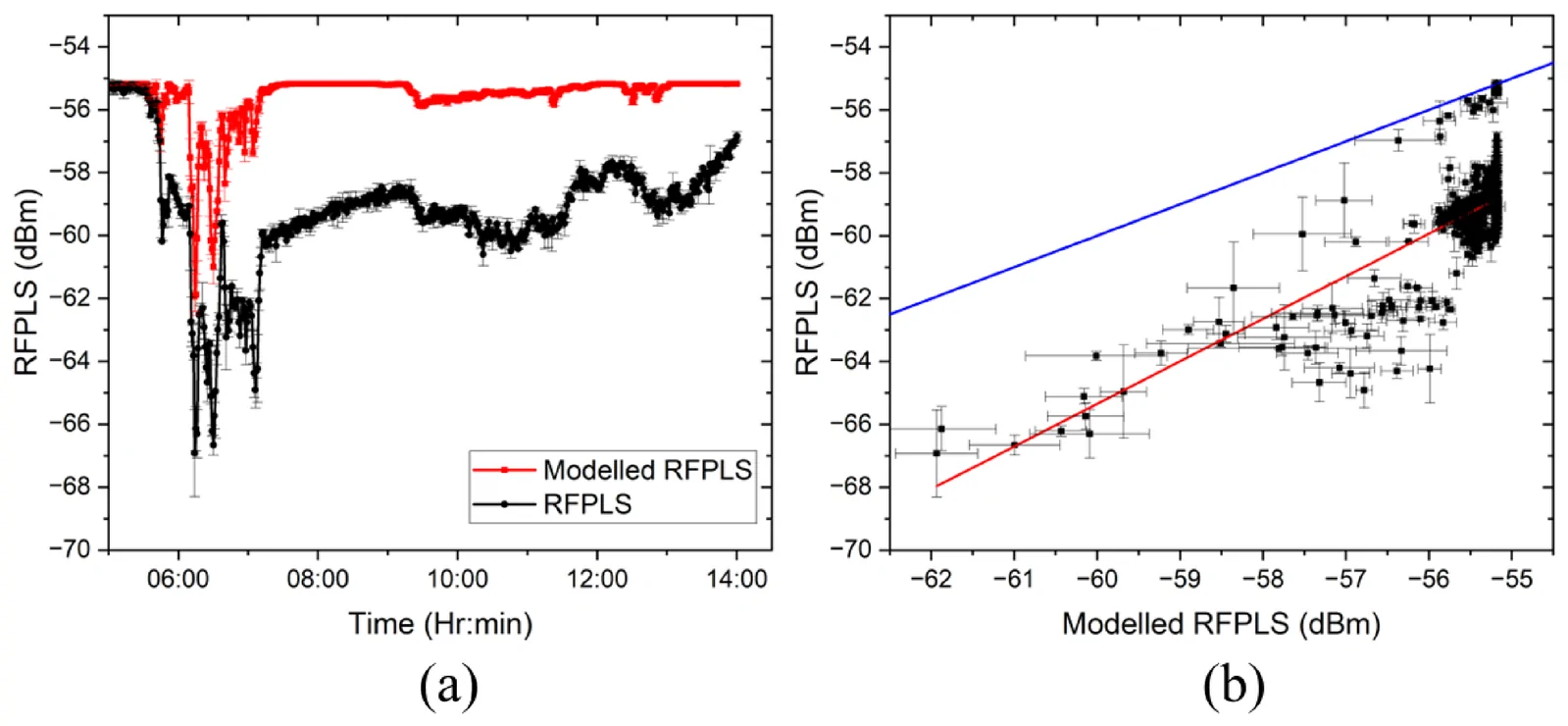
(**a**) The modelled RF path loss signal strength in dBm based on the 77 GHz attenuation coefficient calculated from raindrop size distribution during the 20 October rain event compared to the measured RF path loss signal strength over the same time and (**b**) the modelled signal strength versus the measured signal strength. A guideline (blue) has been provided to show the x = y line on which all points should lie along if the modelled data accurately predicted the measured data. A linear fit of the data is shown in red.

The 77 GHz attenuation coefficient can be used to model the attenuation experienced by both the radar signal travelling to and from the target to the sensor and the RF path loss signal travelling from transmitter to receiver. This modelled data is compared to the measured attenuation in Figure 10 for the radar signal and Figure 11 for the RF path loss signal.

In Figure 10 the modelled data correlates well with the measured radar signal strength data though it appears to understate the peak of the attenuation in the heaviest rain and somewhat underpredicts the signal reduction during the end of the first rain event and throughout the course of the second, much lighter, rain event. Possible explanations are that the DSD-derived 77 GHz attenuation does not include the hail contribution and unmodelled surface wetting effects would remain for a time after the rain events ended.

The modelled RF path loss, as shown in Figure 11, significantly underpredicts the measured RF path loss signal. From this and the more successful radar prediction it can be surmised that whilst the RF path loss measurement clearly ‘sees’ the rain event, the RF path loss signal is dominated by other factors. Surface wetting/ground clutter may go some way to explaining this result as the RF path loss system was contained within a box to protect it from weather damage. This means that the signal would need to be transmitted through two surfaces with possible wetting effects persisting for some time after the rain event, accounting for the much slower recovery of the RF path loss signal after the rain events than predicted by the modelled data.

#### 4.1.3. RADAR: Fog

**Figure 12:**
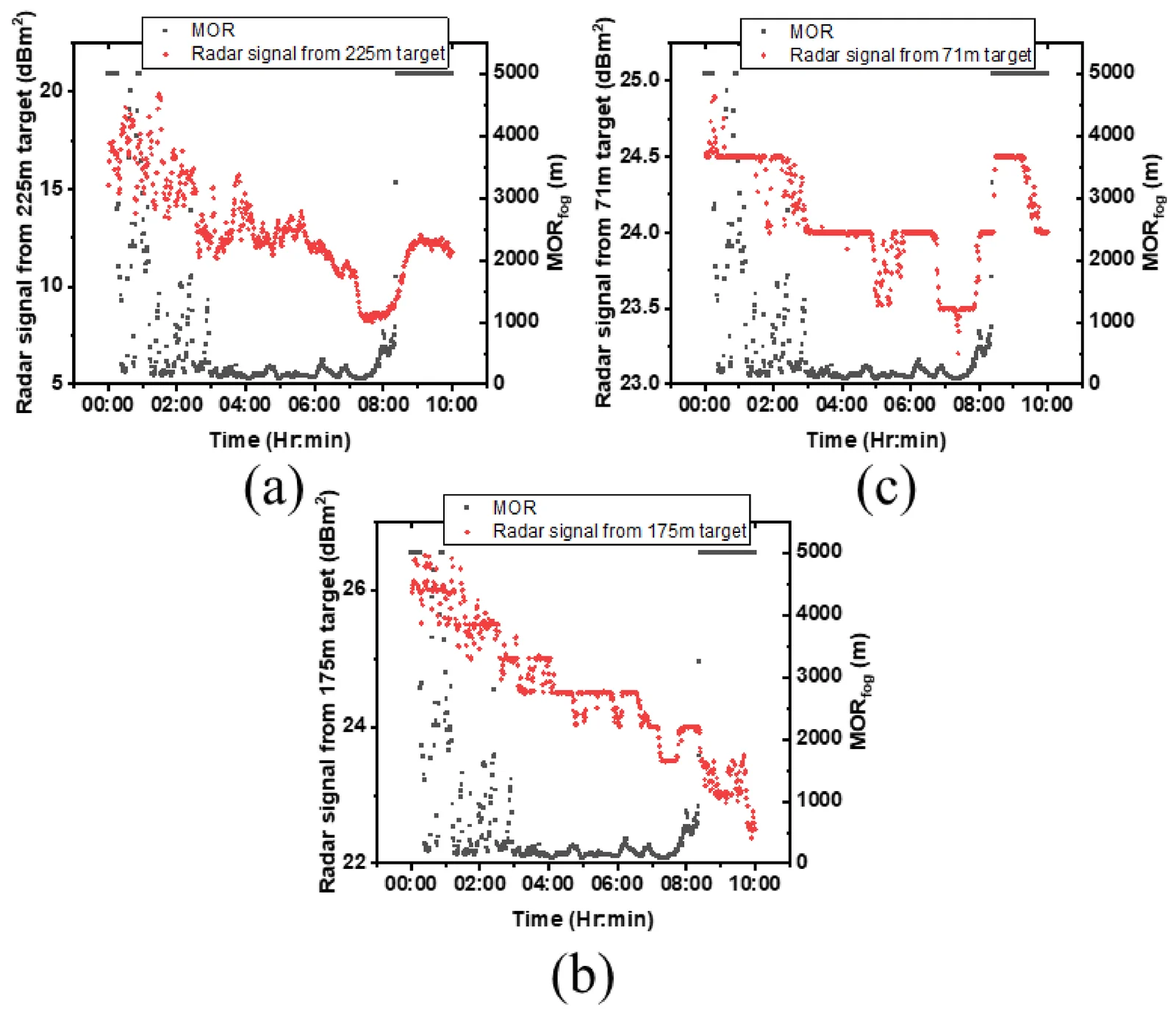
Radar signal over the course of the fog event compared the MOR values for three different corner reflector targets positioned (**a**) 225 m, (**b**) 175 m and (**c**) 71 m away from the radar sensor.

**Figure 13:**
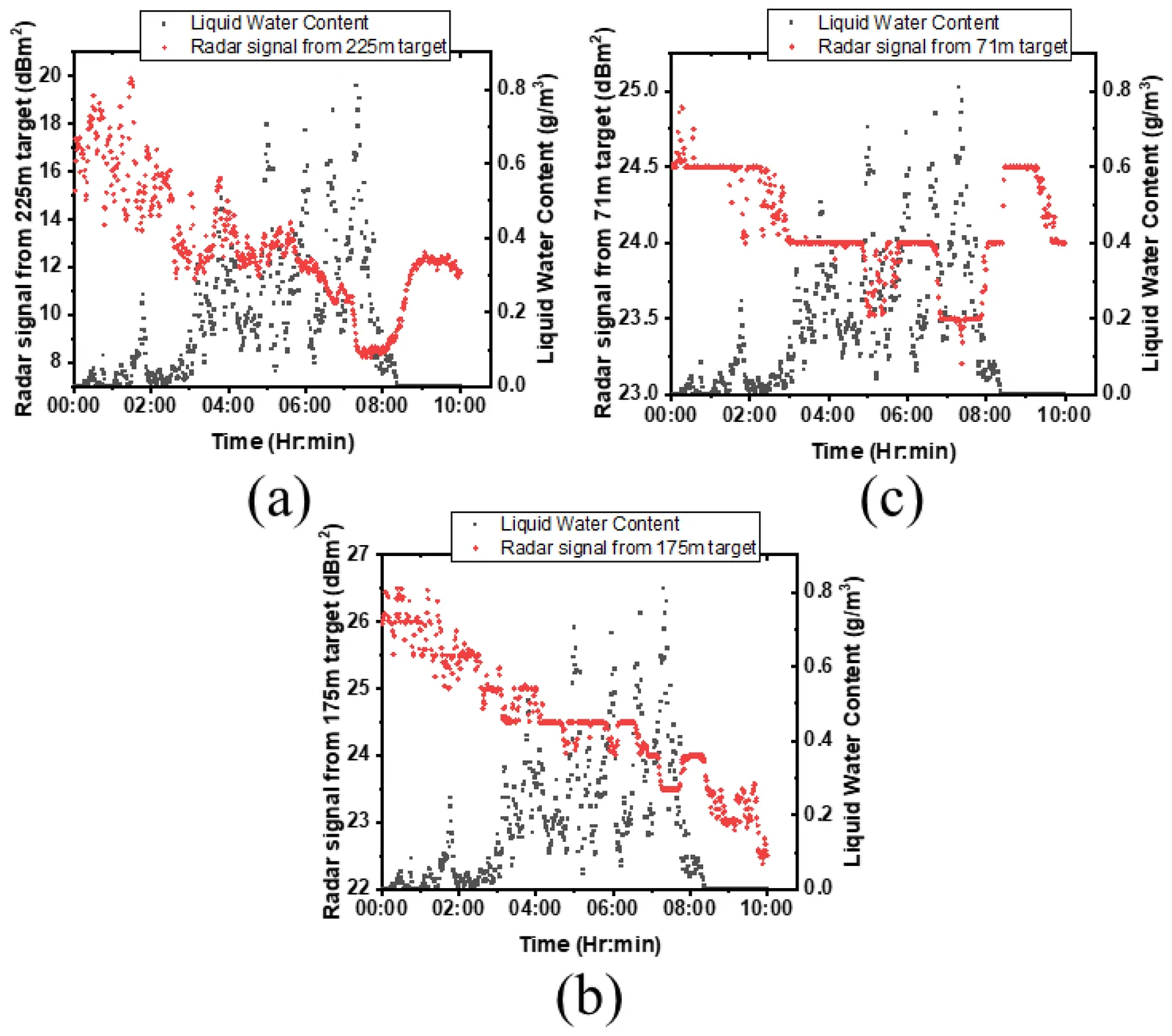
The radar signal over the course of the fog event compared to the liquid water content values for three different targets positioned (**a**) 225 m, (**b**) 175 m and (**c**) 71 m from the radar.

Radar returns from targets at 71, 175 and 225 m range are presented during a fog event on the morning of 18 March 2022. Figure 12 presents radar signal compared to the MOR value and liquid water content in Figure 13.

The MOR value in Figure 12 remains consistently low throughout the fog event from around 03:00 am whilst the radar signal degrades over the course of the morning. The signal from targets at 225 m and 71 m recovers after the fog event ends unlike the signal from the 175 m target which remains low. This continuous reduction in the radar signal throughout the fog event is attributed to the build-up of fog droplets (or condensation from the saturated atmosphere) on the target and sensor surfaces. In Figure 13, unlike the MOR value which remains consistent, the liquid water content (LWC) shows significant variation throughout the fog event. This is not unexpected as the MOR and LWC respond very differently to the size distribution of the fog droplets. Also, the FM120 fog spectrometer does not resolve droplets smaller than 1 µm diameter, which may reduce visibility while not contributing significantly to LWC.

**Figure 14:**
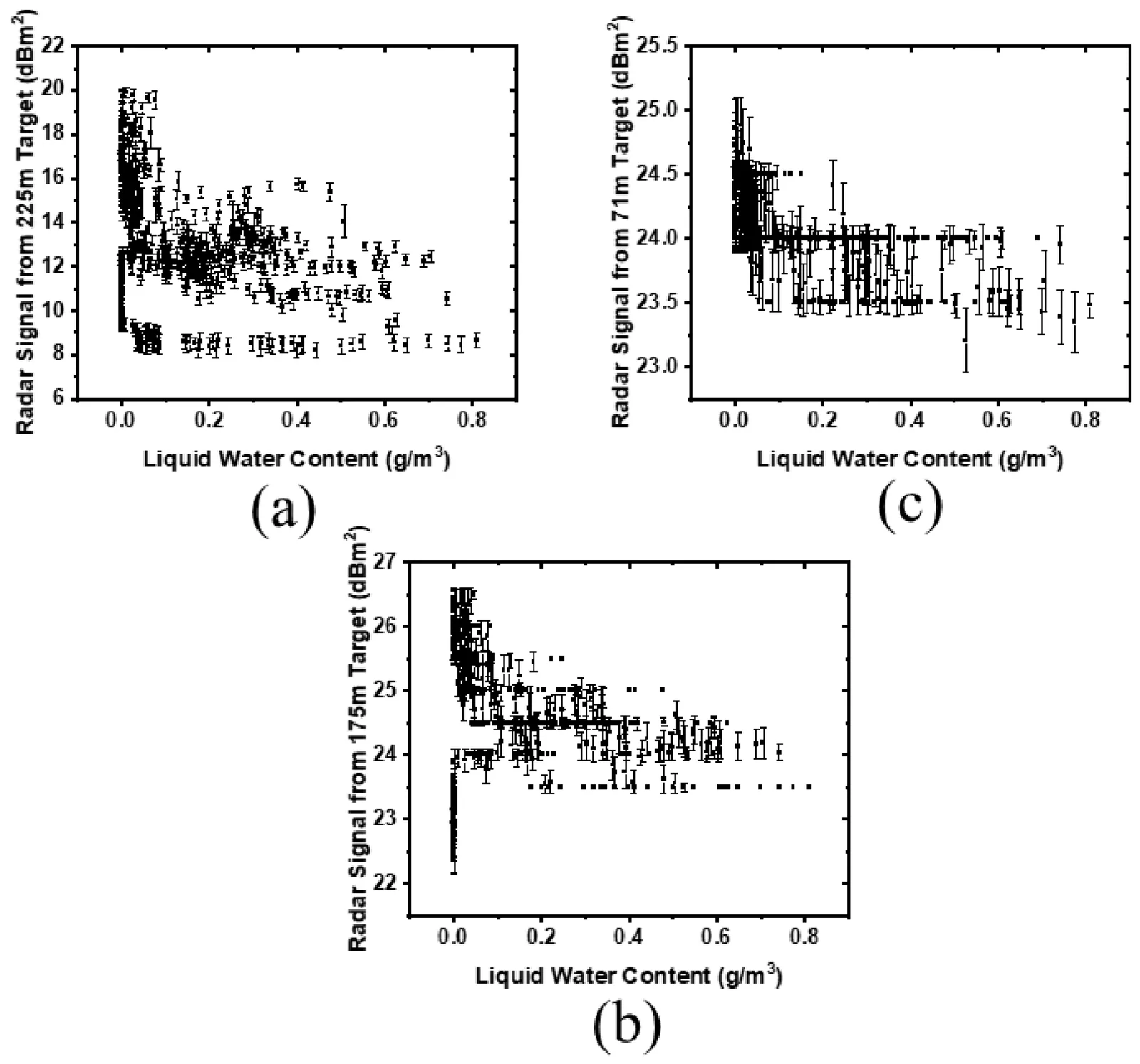
Radar signal from (**a**) the 225 m target, (**b**) the 175 m target and (**c**) the 71 m target compared to the liquid water content values during the fog event.

At the wavelength of the radar and RFPLS, the complex refractive index of water and small fog droplet sizes mean that the path attenuation, which is dominated by absorption rather than scattering, would be expected to correlate well with the LWC (and significantly better than with MOR) and these values have been directly compared for the three radar targets in Figure 14 below. For all three targets, there is a general negative correlation between the radar signal and the liquid water content values. However, there is a significant amount of variation in the radar signal at low liquid water content values, indicating that the amount of liquid water suspended in the air is not the only determinant of radar signal degradation.

### 4.2. LiDAR Measurements

Raw LiDAR data contains a Cartesian three-dimensional point cloud with each point having a reflected intensity, e.g., Point_n_ (x, y, z, intensity). In a similar method to radar analysis, noise is removed by intensity filtering, and each target is isolated through a three-dimensional bounding box centred on the target’s known location. The number of points and weighted mean intensity within the bounding box create a dataset over the period for comparison with weather parameters. For a given target, a reduced point-cloud number typically signifies lower visibility and is a potential source of higher uncertainty in reflectivity value.

It is important to note that the trends seen below may not be generalised to all objects seen by the LiDAR. Many other factors can influence not only the levels of degradation, but the type of physical effect that is causing a change in a target’s observed properties. Factors such as the material, shape or size of the target can cause the alterations mentioned. Also, positional variations such as the target’s distance and relative position in the LiDAR’s internal coordinate system can affect how the environmental changes interact with the resulting data. This is inherently seen when comparing the data from the Sensor Assurance Framework (SAF) data portal (https://saf.npl.co.uk/ (accessed on 28 August 2026)) with the results seen below in this paper. Due to the evolution of the testbed over time, the type of targets along with their positions changed, making direct comparison of this early data and the available portal data not straightforward.

#### 4.2.1. LiDAR: Flat Weather

**Figure 15:**
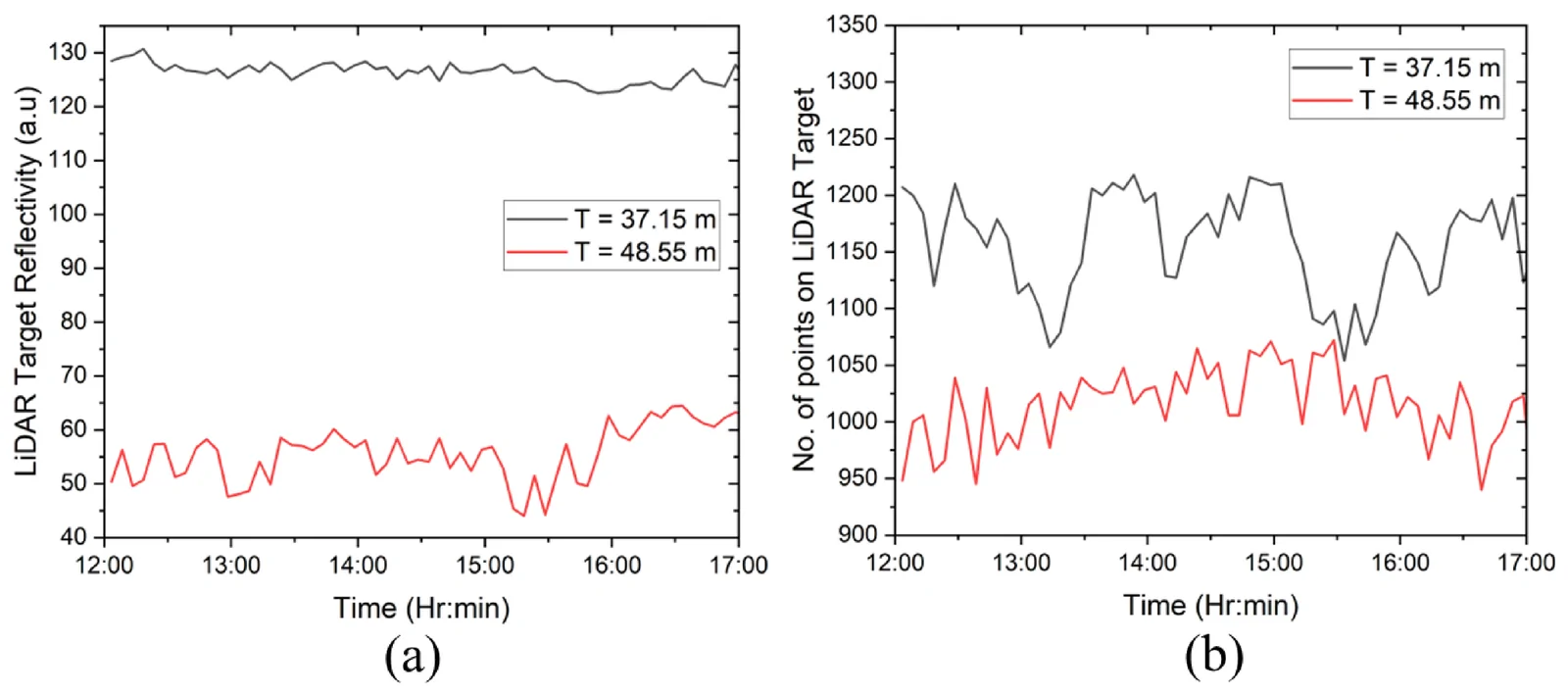
LiDAR target (**a**) reflectivity and (**b**) total number of point-cloud points for 13 September 2022 for targets at 37.15 and 48.55 m.

The reflectivity and point-cloud number for two LiDAR targets are presented for 13 September 2022, a ‘flat’ weather day, in Figure 15. The stable atmospheric conditions reveal that the baseline noise for our LiDAR observations is a few percent for near-range high-brightness targets, falling to 10% for targets in the far range. Specifically, the mean and standard deviations for reflectivity were 126 ± 2 a.u (target at 37.15 m) and 56 ± 5 a.u (target at 48.55 m). The mean and standard deviation of cloud points were 1160 ± 40 (target at 37.15 m) and 1010 ± 30 (target at 48.55 m).

#### 4.2.2. LiDAR: Rainfall

**Figure 16:**
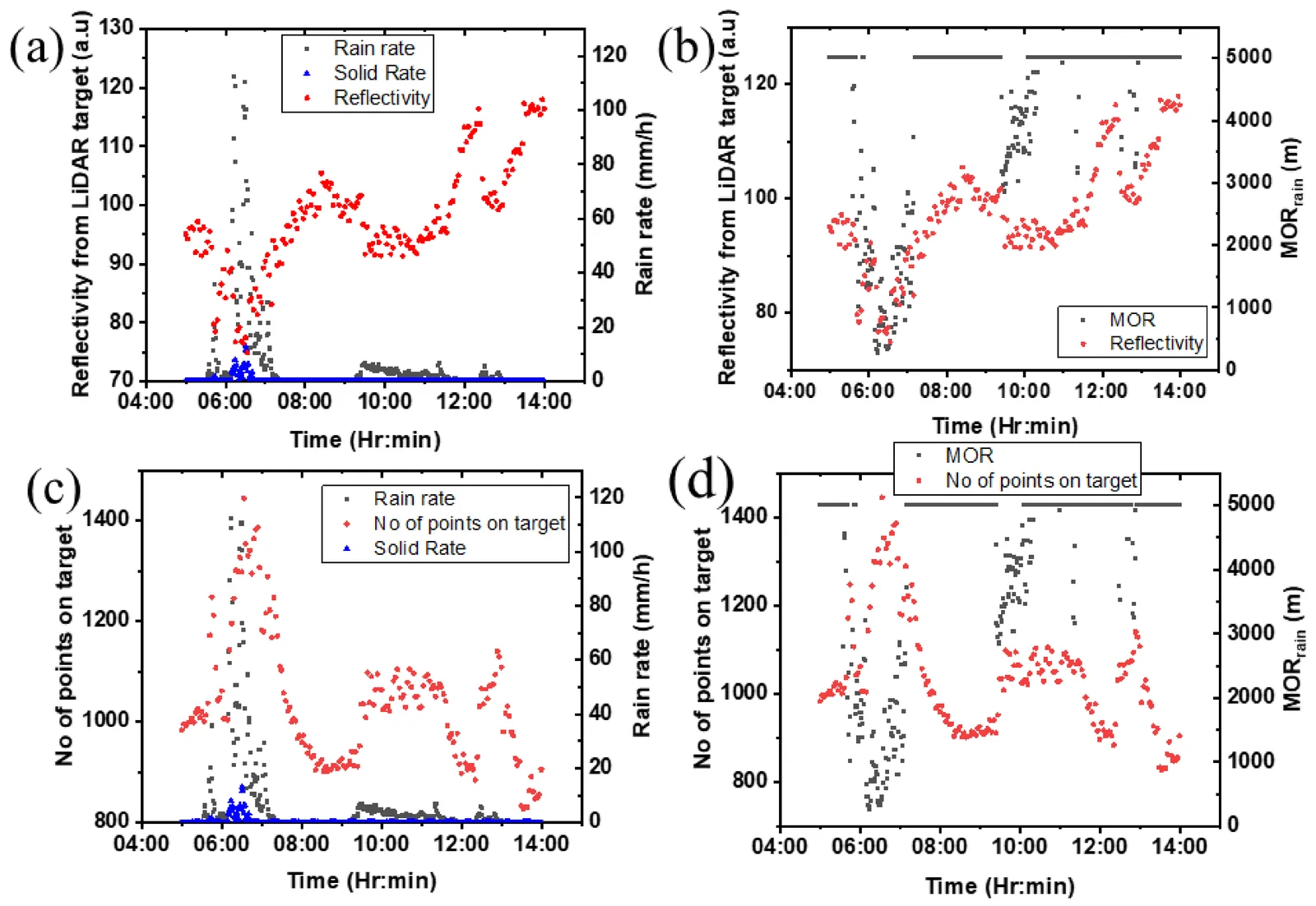
Average reflectivity of a target vs. (**a**) rain and solid rate and (**b**) MOR and the number of points on the target vs. (**c**) rain and solid rate and (**d**) MOR.

The LiDAR target reflected signal and number of points for the 20 October 2022 rain event are compared to the rain (and solid) rate and MOR values in Figure 16. The target reflectivity decreases during the short, high-intensity rain event and then recovers. Conversely, the number of points of the target increases during the rain event. The decrease in reflectivity can be explained by a combination of Mie scattering through the raindrops and any accumulation of droplets on the target and sensor window surface. The increased point number is possibly from an increase in low-level scattering from the target surface, resulting in more target points with a lower average reflectivity.

**Figure 17:**
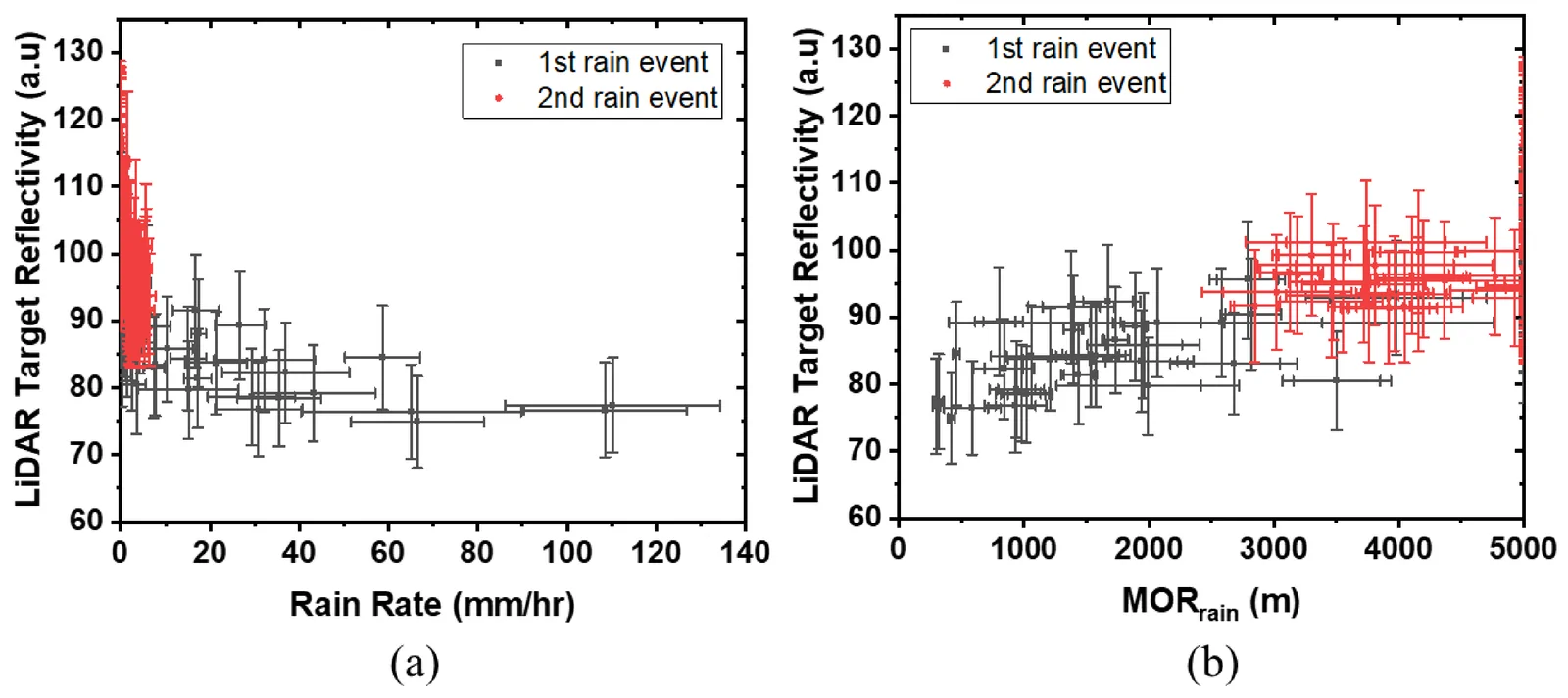
LiDAR reflectivity during the 1st (heavy) and 2nd (light) rain events compared to (**a**) the rain rate and (**b**) the MOR. Error bars for LiDAR reflectivity are calculated from the variation in sensor performance when not exposed to any significant weather events; rain rate and MOR error bars have been calculated based on site-wide variation in drop size distribution measurements.

Direct comparisons of the LiDAR target reflectivity, rain rate and MOR values during the course of the two rain events on 20 October 2022 are presented in Figure 17. Error bars for the LiDAR reflectivity signals have been calculated from the flat weather data as (sd_flat_/mean_flat_) × reflectivity_rain_. It should be noted that this error represents the uncertainty in the general performance of the sensor when not exposed to any significant weather events and any uncertainty in the analysis procedures used to extract the target reflectivity data from the raw saved data. It does not account for any additional uncertainty in the sensor performance caused by the rain event, for example accumulation of droplets on the sensor window or fluctuations in sensor temperature.

These plots show that the LiDAR reflectivity decreases with increasing rain rate and decreasing MOR during the first rainfall event with high rain rates. However, the reflectivity does not appear to have a strong correlation with either rain rate or MOR during the second, much lighter, rain event.

**Figure 18:**
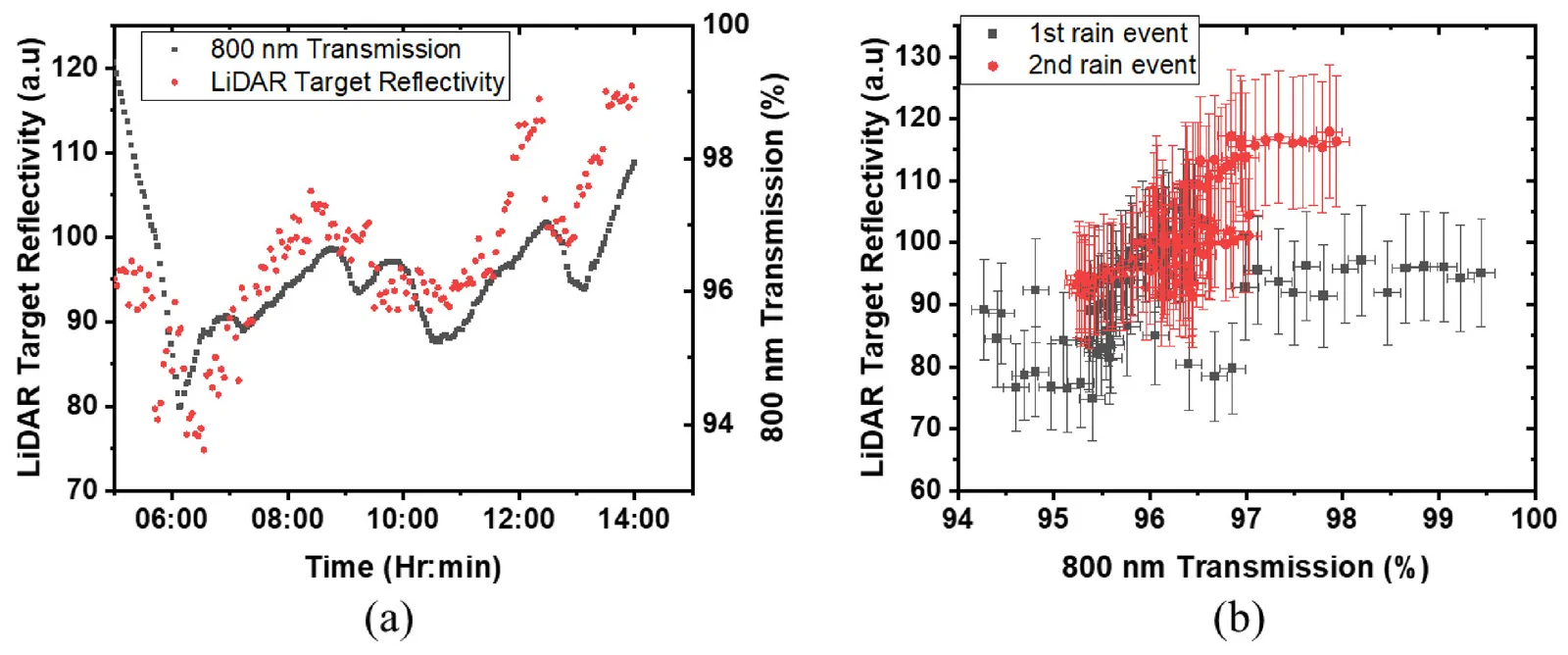
(**a**) Time series plots comparing the LiDAR target reflectivity with the 800 nm transmission percentage as measured by the optical path loss system. (**b**) Direct comparison of these two variables with associated error bars.

Alongside the meteorological data, the LiDAR performance data can also be compared to the optical path loss system data, which gives the percentage transmission of light at several different wavelengths during the weather events. Figure 18 shows a comparison of the LiDAR target reflectivity with the 800 nm percentage transmission as measured by the OPLS. Error bars for the OPLS data have been calculated using the mean and standard deviation from OPLS data from a ‘flat’ weather day as has previously been done for the LiDAR reflectivity data. The 800 nm data have been chosen as this is the closest available wavelength to the LiDAR operating wavelength (~900 nm). Figure 18a shows that the reflectivity and 800 nm transmission follow along the same general trends as one another, increasing and decreasing at approximately the same time. Figure 18b shows a somewhat positive correlation of the 800 nm percentage transmission and the target reflectivity but an increase in attenuation (resulting in a reduction in transmission) at similar wavelengths is insufficient alone to explain the behaviour of the LiDAR target reflectivity.

#### 4.2.3. LiDAR: Fog

**Figure 19:**
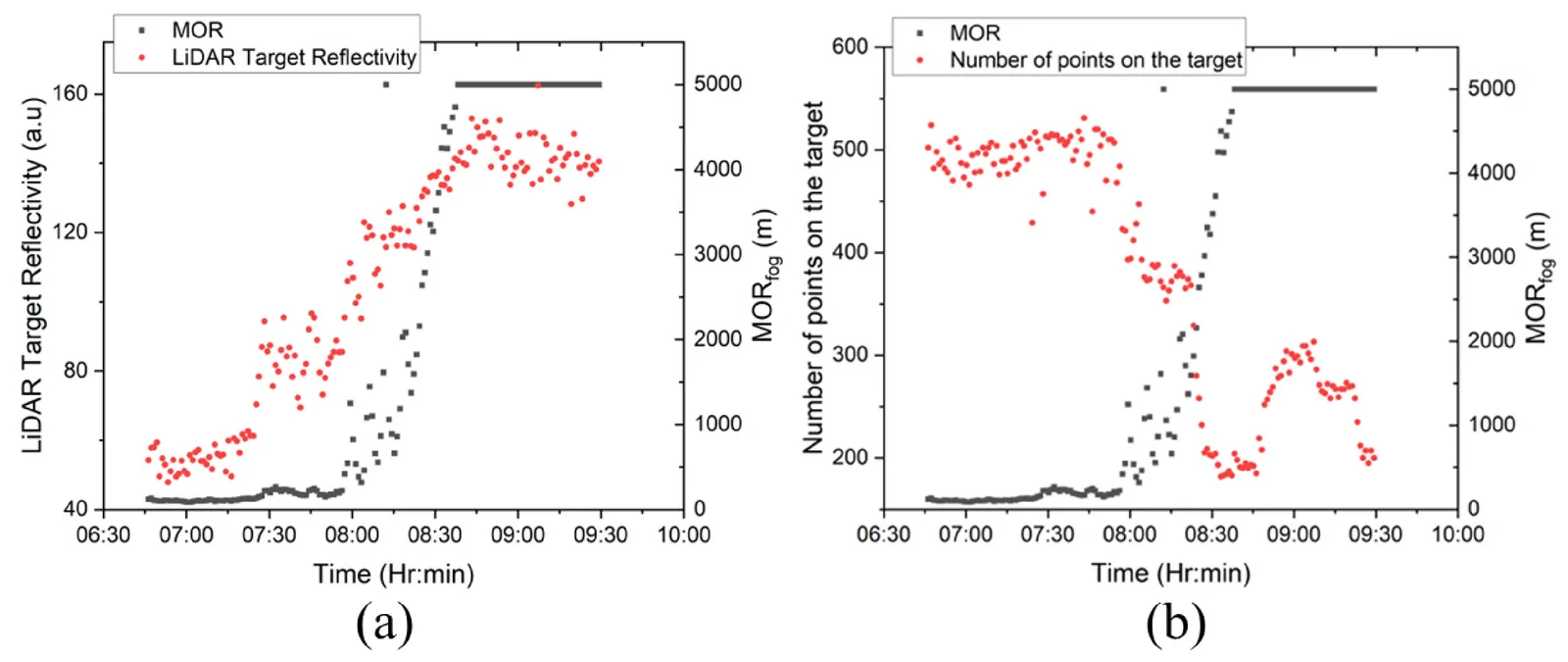
MOR over the course of the morning of 26 August during the fog event versus (**a**) the average reflectivity from points on the LiDAR target and (**b**) the number of points on the target.

**Figure 20:**
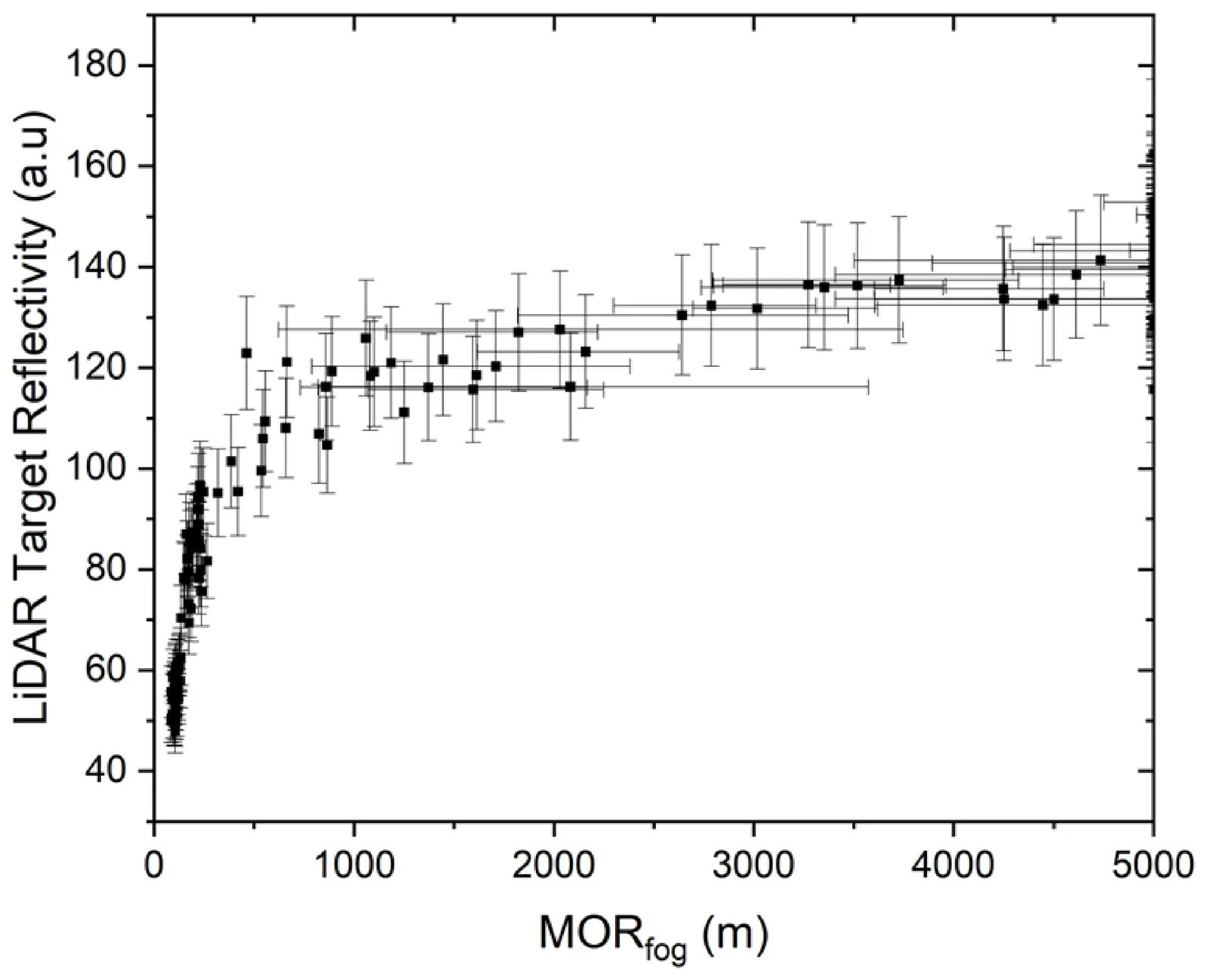
Comparison of the LiDAR target reflectivity and the MOR during the last two hours of the fog event on 26 August 2022.

LiDAR signal during a fog event on the morning of 26 August 2022 has been compared to the MOR values from Met Office visiometers. Liquid water content data for this fog event is not available due to a power outage to this section of the testbed. Time series plots comparing the target reflectivity and number of points on the target are shown in Figure 19 and a direct comparison of the LiDAR target reflectivity and the MOR is provided in Figure 20. The MOR-versus-LiDAR-reflectivity plots shows that the reflectivity is low during the fog event when the MOR is also low and increases when the MOR increases as the fog begins to disperse. Conversely, the number of points associated with the LiDAR target, shown in Figure 19b, is high when the MOR is low and decreases when the visibility improves. This may also be due to the wetting of the target surface during the fog event causing additional scattering compared to the dry target, creating more associated points. It should also be noted that there is another increase, and subsequent decrease, in the number of points on the target after the fog has dispersed and the visibility is consistently high; this indicates that another factor may be playing a role in the behaviour of this property.

The direct comparison of the LiDAR target reflectivity and MOR values in Figure 20 shows a strong positive correlation between these two properties for MOR values < 750 m which plateaus past this value. It should be noted that Figure 20 does not give any error bars on the MOR values for the lower visibility data points due to the power outage that caused two of the three visiometers to cease recording, meaning that only one value for MOR exists for each one-minute time interval.

### 4.3. Camera

Testbed cameras create pixel images of the test targets which are converted to SFR values using the methods described in Section 2.1. SFR target values, as with radar and LiDAR, are plotted with respect to the corresponding weather conditions to observe correlations.

#### 4.3.1. Camera: Flat Weather Conditions

**Figure 21:**
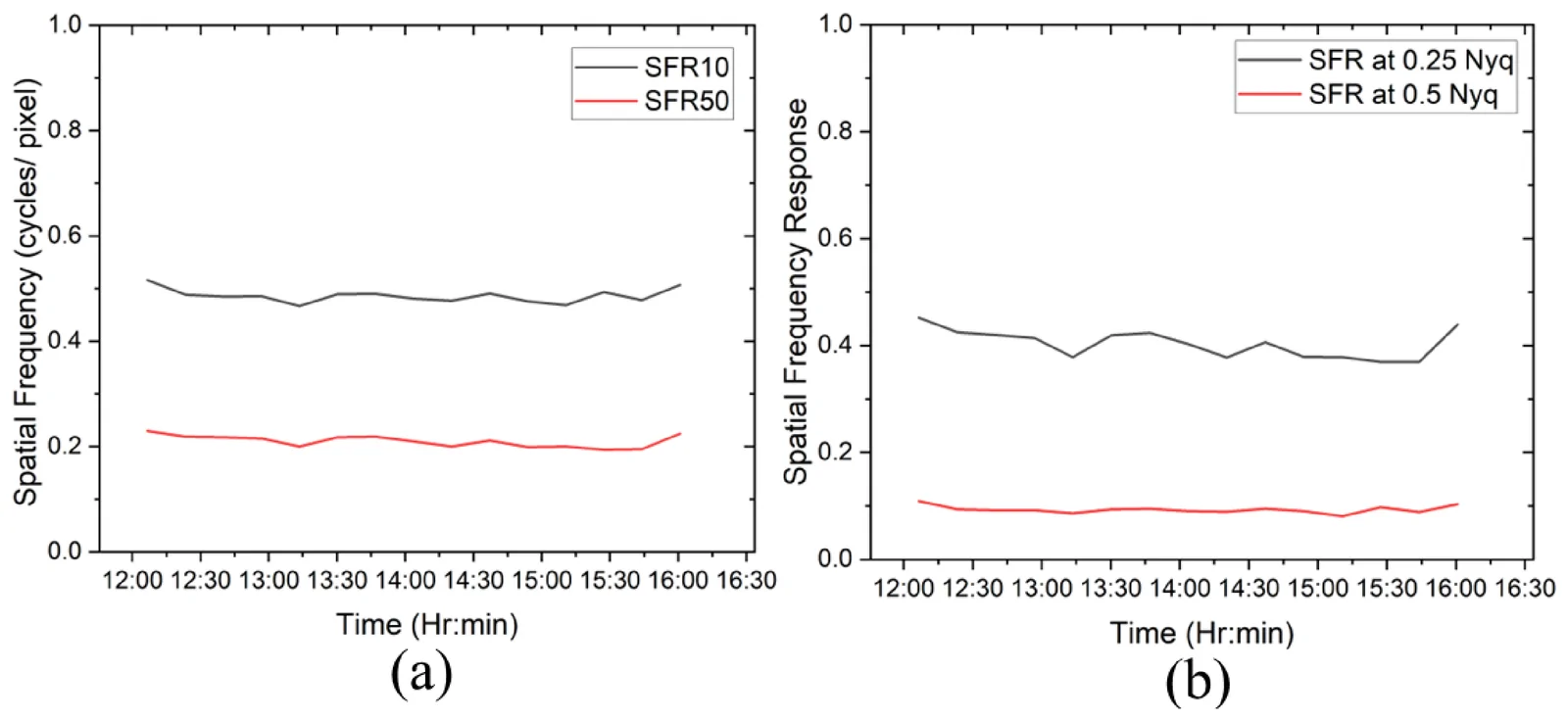
Camera observation of flat weather day: (**a**) the spatial frequencies for SFR10 and SFR50 and (**b**) the spatial frequency response at 0.5 and 0.25 Nyquist values over a four-hour period on 13 September 2022, a ‘flat’ illumination day with no significant weather events.

Four SFR-derived values are presented in Figure 21 between the hours of 12:00 and 16:00 for the ‘flat’ weather day on 13 September 2022 with small variations over the time period. The mean and standard deviations represent the inherent errors of the system and algorithms and are 0.49 ± 0.01 cycles/pixel (SFR10), 0.21 ± 0.01 cycles/pixel (SFR50), 0.40 ± 0.03 (SFR at 0.25 Nyq) and 0.093 ± 0.007 (SFR at 0.5 Nyq).

#### 4.3.2. Camera: Rainfall

**Figure 22:**
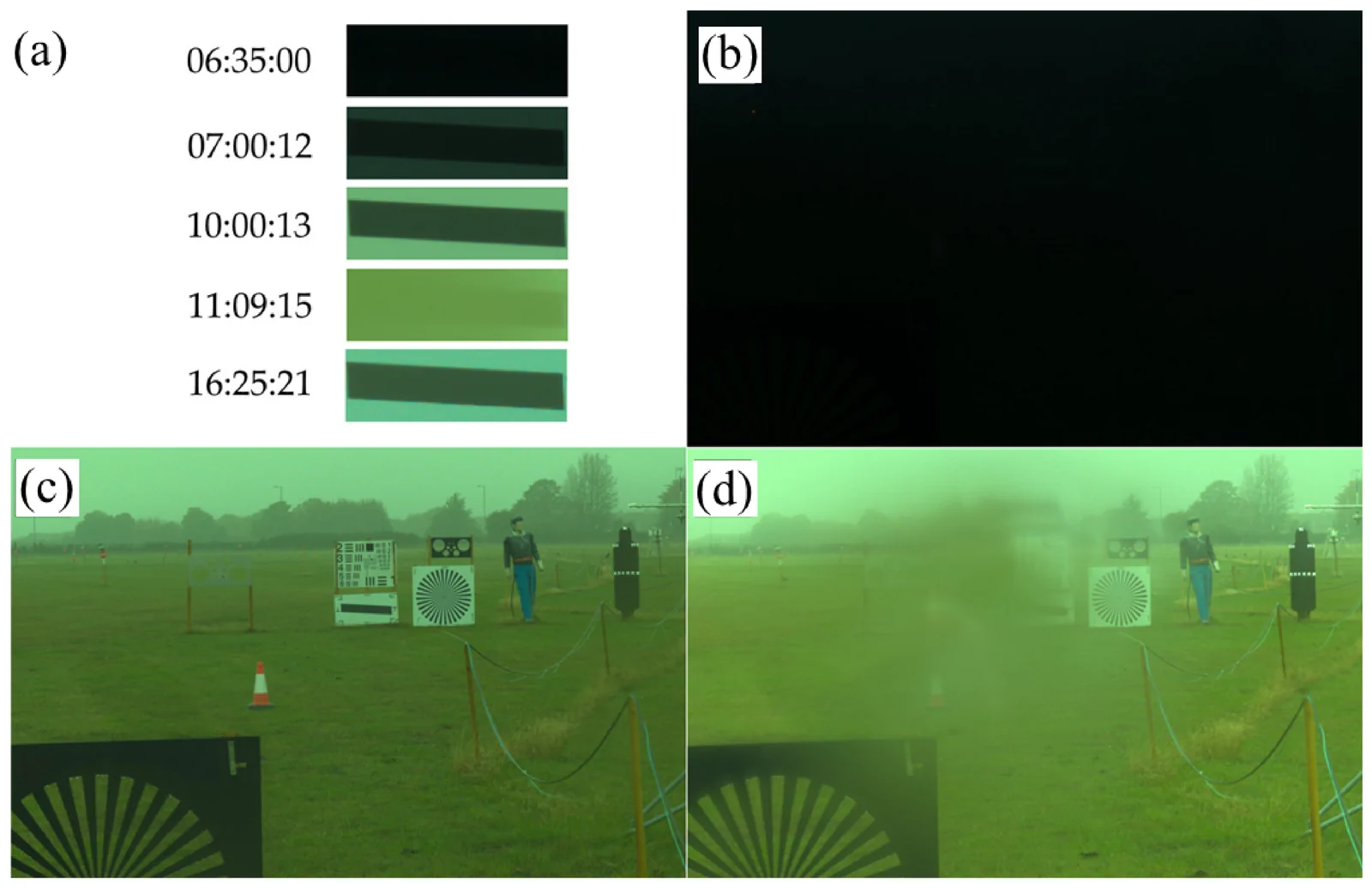
(**a**) Edge target shown at five times during the day of 20 October 2022. Full-frame images at (**b**) 06:35:00 (close to sunrise), (**c**) 10:00:13, and (**d**) 11:09:15. In (**d**) a water droplet on the window to Camera 1’s housing obscures part of the field of view. The green hue in the images arises from the camera’s auto white balance function.

**Figure 23:**
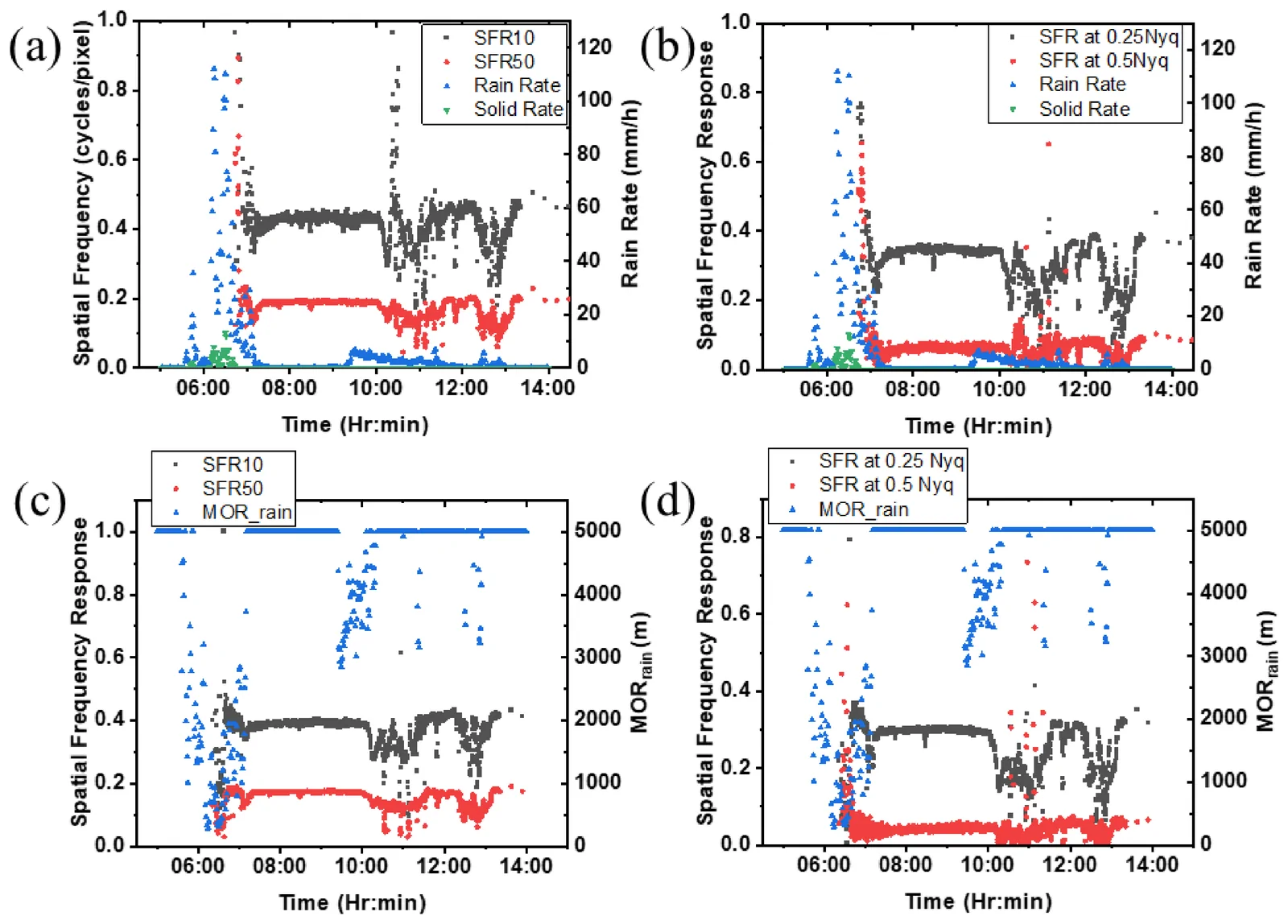
Camera 20 October time series plots for SFR10 and SFR50 during the rainfall compared to (**a**) the rain/solid rates and (**c**) the MOR and the SFR values at 0.25 and 0.5 Nyquist compared to (**b**) the rain/solid rates and (**d**) the MOR.

A sequence of camera images from the heavy rainfall event on 20 October 2022 of the edge target is shown in Figure 22a, showing how its appearance to the camera changed the day before, during and after the rainfall. Images of the camera’s full field of view are shown in Figure 22b–d. Sunrise did not occur until after 6:30am and thus target visibility was low during the event’s heaviest phase due to low light levels rather the rain itself (Figure 22b). During the lighter second rain event, shown in Figure 22c,d, the field of view was affected greatly by the presence of water droplets on the camera housing window. The rain rate graphs in Figure 23 show that all the SFR-derived values are stable during the periods between rain events. During the second rain event, which had a significantly lower rain rate than the first, the SFR-derived values remained consistent for approximately the first half hour of the rainfall before becoming unstable. This coincided with multiple instances of water droplets forming on the camera housing window.

**Figure 24:**
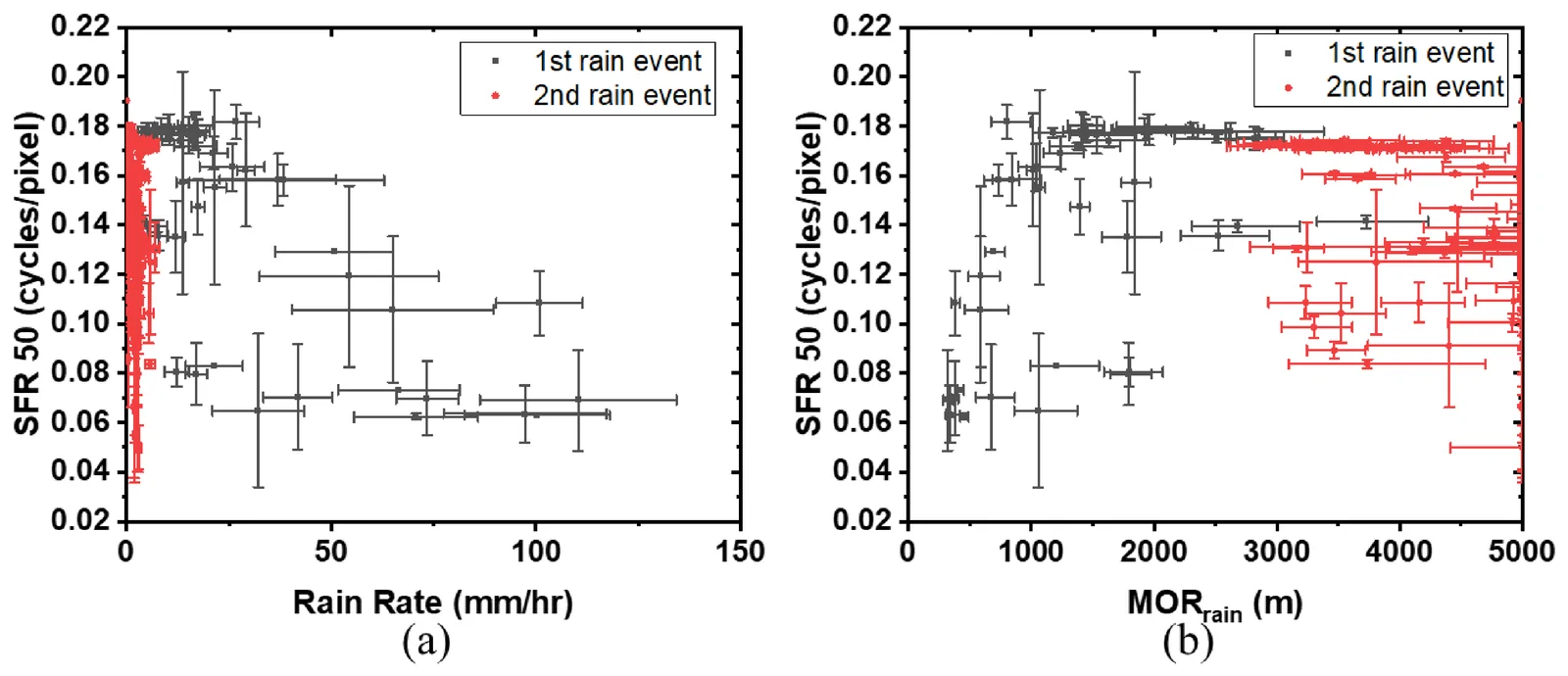
Comparison of the spatial frequency value at SFR50 with (**a**) the rain rate and (**b**) the MOR values.

The relationship between SFR50, rain rate, and MOR is shown in Figure 24. Points within the plots are separated by colour to indicate the date’s first (black points) and second (red points) rain events. Figure 24a shows the first rain event produces a negative correlation with increasing rain rate indicating degradation of imaging performance, a trend that is not seen during the second event (red points). The second rain event, consisting of lighter rain rates less than 7 mm/h spread across several hours, produced a wide spread of SFR50 values, at times reducing the SFR50 value to below that caused by heavier rain rates during the first rain event. The large observed variation in SFR50 was caused by water droplets on the camera housing’s window (Figure 22d) rather than rain falling through the atmosphere. Comparing the relationship between SFR50 and MOR during the first rain event in Figure 24b shows a positive correlation between 0 m and 1000 m where imaging performance increases with visibility. Once MOR increases beyond 1000 m, SFR50 plateaus to its maximum value. The plateau continues into the higher MOR values during the second rain event, albeit with large scatter in SFR50 due to the aforementioned water droplets on the camera housing window.

#### 4.3.3. Camera: Fog

**Figure 25:**
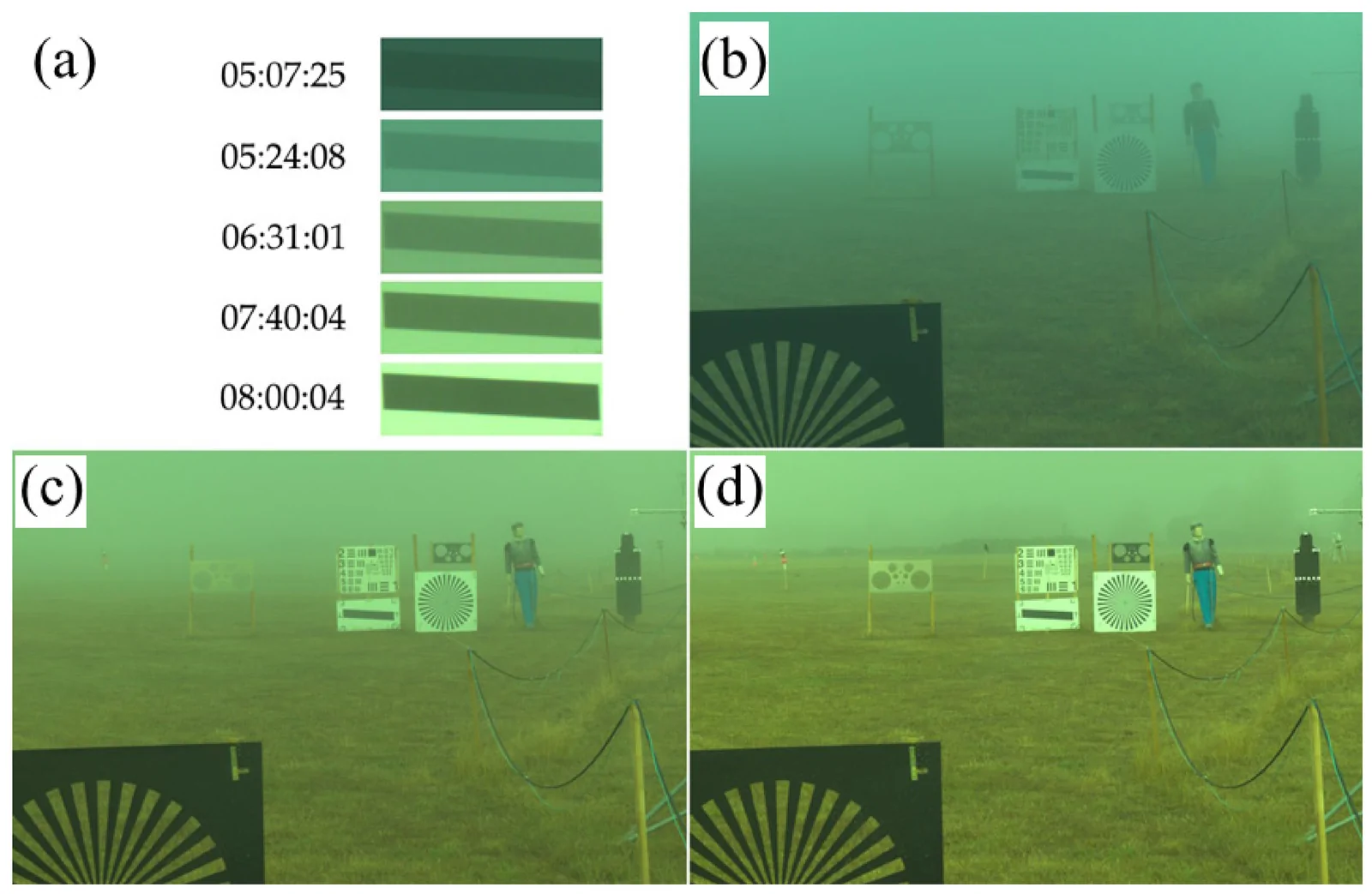
(**a**) The slanted edge target shown at five different times during the day of 26 August 2022. Images from Camera 1 taken at (**b**) 05:24:08, (**c**) 07:40:04, and (**d**) 08:00:04. The green hue in the images arises from the camera’s auto white balance function.

**Figure 26:**
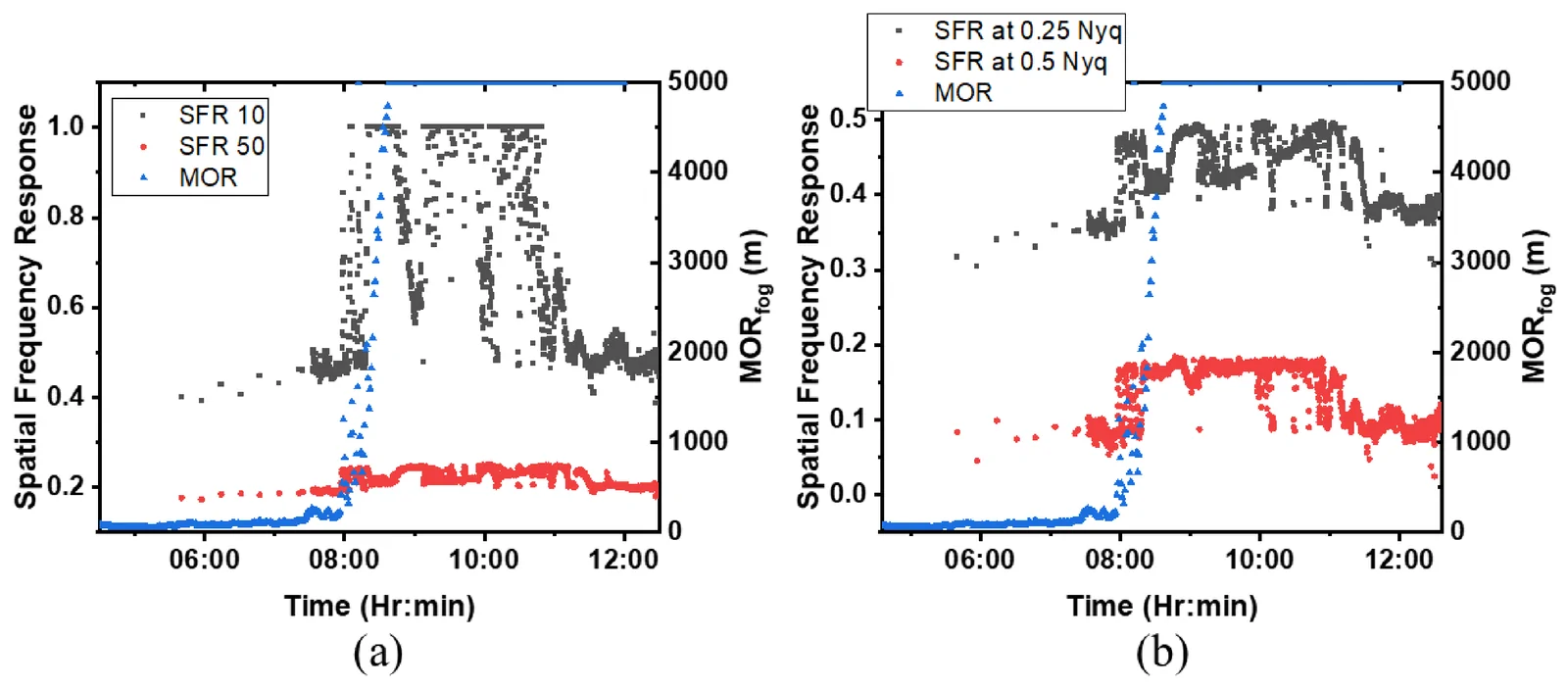
MOR values from the last two hours of the fog event on the morning of 26 August compared to (**a**) the SFR10 and SFR50 values and (**b**) the SFR values at 0.25 and 0.5 Nyquist.

The camera view of 26 August 2022 during an overnight fog event of the edge target is shown as a sequence of images in Figure 25a, and of the full field of view in Figure 25b–d. The SFR and MOR values for the period are shown in Figure 26, where sunrise occurred at 5:00 and the fog dissipated from the targets by 8:00. The MOR values after sunrise increase as the fog disperses and the SFR-derived values also show a small increase before becoming unstable as the camera began overexposing the white areas of the target as the ambient light levels varied.

## 5. Discussion

The case studies in the previous sections provide a demonstration of the data produced by the testbed’s multi-year operation to characterise sensor performance when exposed to rain and fog. In both weather cases radar, LiDAR and camera performance were degraded compared to the ‘flat’ weather days; however, the amount of degradation experienced was dependent on both the severity of the weather event and secondary effects during/after the weather event, such as target or sensor window wetting. These secondary effects are not straightforwardly measurable and, indeed, during the on-road operation of the AV they will generally not be known and will also be challenging to represent comprehensively in simulation environments. Furthermore, in many cases these secondary weather effects continued to affect sensor performance after the weather event had ended. This was particularly noticeable after the heavy rain event, when all three sensor types took time after the weather event had ended to recover to their performance level prior to the rain event. This is likely due to sensor window/target wetting which would affect sensor performance until after they had dried. The exact time for recovery varies between the measured sensors and this is likely due to a mixture of variables including the sensor temperature and the physics of how the relevant wavelengths of light interact with droplets. This suggests that there are practical limits to which the total impact of the weather on sensor performance can be both quantified and represented in an ODD and predicted from knowledge of the Current Operational Domain (COD) in operations. This will demand a rigorous handling of uncertainty in expressing these ODD (and COD) parameters and careful consideration of how the determination of an AV being within its ODD is made in real time.

For radar, the returned signal shows a broadly linear decrease with increasing rain rate and could be somewhat successfully predicted from the 77 GHz attenuation coefficient calculated from the raindrop size distribution measured by the Met Office disdrometers. There was a small underestimation of the loss experienced by the radar signal using this model that is likely to do with secondary weather effects. From the data taken on the fog example day, the degradation to the radar signal was compared to both the MOR and the fog liquid water content. It can easily be seen from this comparison that the behaviour of the radar signal during fog cannot be adequately explained by a variation in either variable alone. This might be reasonably expected in the case of the MOR, which is ultimately a measure of attenuation at visible wavelengths. At radar wavelengths the attenuation due to small droplets (which, unlike raindrops, are considerably smaller than the wavelength) is proportional to liquid water content and so the observed behaviour must be largely down to secondary effects. The testbed has captured several more fog events during its two-year operation and understanding the behaviour of the radar on those days forms part of the future analysis work that will be performed on this very large dataset.

For LiDAR, target reflectivity reduced during both rain events, but the point-cloud density showed a small increase with rain attributed to target surface wetting. During the fog event on 26 August, the target reflectivity and number of target points follow similar trends, reflectivity decreasing and target points increasing, again likely due to the target wetting, although we note that this was highly dependent on target type and viewing geometry. Reduced reflectivity from the targets translates in real terms to a reduction in the apparent range of the LiDAR view. The increase in targets is a somewhat unexpected trend but could be due to increased scattering on the targets’ surface caused by water droplets or condensation.

For cameras, during the example rain event SFR-derived values showed a negative correlation with rainfall rate and positive correlation with MOR up to 1000 m. A positive correlation was also observed between the SFR-derived values and MOR measured during the example fog event. However, water droplets on the camera housing window could cause decreases in the SFR-derived values of the same magnitude as heavy atmospheric rainfall and low MOR, even when the actual rainfall rate was low and MOR was high. This illustrates that the degradative effects of hydrometeors in the atmosphere between the target and sensor can be secondary to the degradative effects caused upon the sensor itself and the importance of cameras requiring clear and unobscured fields of view to have their best operational performance. With degraded SFR, a camera would not be able to resolve finer details within a scene, potentially causing an AV to not perceive or identify hazards and vulnerable road users, especially those at a distance.

Overall, data for each sensor and weather event demonstrates a high level of complexity between sensor performance and conventional weather parameters such as rainfall rate or MOR. For example, a clear trend for radar degradation vs. rainfall rate is shown, but the performance of the LiDAR used here is less predictable. The results highlight not only the need for sensor testing over a wide range of conditions but that the quantification of a weather event cannot utilise a simple or single parameter to ensure accuracy in prediction of degradation. Indeed, the lack of one-to-one mapping between possible ODD/COD weather parameters and sensor performance limits the extent to which an explicit determination of operational limits in weather is possible and necessitates the use of some implicit determination of sensor performance by the vehicle itself.

An additional challenge, demonstrated within this paper, is understanding the appropriate level of uncertainty to apply to measured weather effects when considering the limitations under which an AV can operate. The 20 October rainfall example demonstrates up to ~20 mm/h uncertainty in the testbed 1 min rainfall rate, this coming from both the spatial variability across the site and also the measurement limitations of the disdrometers themselves.

While it is unlikely that this uncertainty would be materially reduced by the addition of more disdrometers (the measurements presented in this paper representing “as good as it gets” for an outdoor test site), it is equally true that in an operational context such a density of measurement is unlikely to be achieved and the current best ‘guaranteed’ coverage, largely fed by operational radar networks, would be considerably coarser (and of lower accuracy). These constraints place strong limits on our confidence in both determining the ODD (on the testbed) and the Current Operational Domain (COD) when the AV is in operation. If we take a simplistic view that our confidence that an AV is within its rainfall ODD limits is proportional to the difference between COD and ODD values, if real-time weather awareness (‘explicit’ determination of the current rainfall) forms part of the AV safety case then, initially at least, a very conservative ODD value must first be adopted to account for the large uncertainty in the COD. For this reason other approaches (such as ‘implicit’ detection of sensor impairment by the AV) may be advantageous. The complexity of measuring rainfall demonstrated on the testbed in this paper also highlights the need for AI algorithm training datasets to consider the variety of rain scenes they include. As indicated in the Introduction, there are available sensor perception training datasets that include weather effects but, for example, the rain scenes may have no further description than ‘light’ or ‘heavy’ and this is insufficient information to determine whether an algorithm trained on this dataset will be able to cope with the full range of ‘heavy’ rain.

The situation becomes increasingly more complex when we remove the assumption that there is a single meteorological parameter (in this case rainfall rate) that would adequately predict the impact of rainfall on each of the primary sensor types. This is not the case because of the highly non-linear and wavelength-dependent impact of different-sized water droplets on sensor performance. In the case of rainfall, additional knowledge on the droplet size distribution shape would be required to reduce ODD uncertainty further. Related to this, the data also demonstrates that different conventional meteorological parameters, for example MOR, responded to more than one weather type (i.e., both rain and fog). The result is that ODD parameters may not be independent of each other. The implication is that when constructing ODDs and applying their recommendations within the COD, sufficient meteorological context is essential to properly interpret any ODD boundary values. It is for this reason that the current ISO 34503 provides additional guidance that MOR is of particular relevance when considering the impact of fog on optical systems.

Finally, the analysis of the testbed data highlights the benefits of making a distinction between measurements made by meteorological instrumentation and the ‘testbed parameters’ that are derived from them. Our testbed MOR value was produced either directly from the visiometer in fog (*MOR_fog_*) or by estimating it from the droplet size distribution measurements from the network disdrometers and FM120 (*MOR_rain_*) in rain. These two approaches were implemented in an attempt to produce the optimum and well-understood value for each weather type. A lesson learned from the creation and operation of the Cardington testbed is that meteorological measurements cannot be taken at face value when used in this context and, where there is a significant AV sensitivity to a given meteorological parameter, care must be taken to define and evaluate measures of uncertainty.

## 6. Conclusions

This paper introduces the SA sensor testbed jointly operated by the National Physical Laboratory and the Met Office. The testbed recorded the performance of the most common sensor types used within AVs: radar, LiDAR and camera. Data from these three sensors was recorded over a period of two years during a large variety of different weather events with comprehensive meteorological data for these weather events measured by the Met Office on site to provide the most accurate localised weather data possible. The paper aims to show the breadth of data produced during the lifetime of this testbed and the sensor performance with weather relations that can be explored from this dataset. To do this, two example days were chosen, one with very heavy rainfall and one with fog, and data from all three sensor types alongside the meteorological data has been presented. All three sensor types show degradation in performance during and, in some cases, after the rain and fog events compared to the mild and cloudy conditions experienced at other points within the example days.

The authors wish to highlight that sensor weather degradation in real-world conditions is complex and challenges remain in modelling even for a reference testbed deployment. Whilst the body of data collected from the testbed over its 2-year operation is extensive, there are limitations to the validity of directly applying this data to the modelling of sensors on an AV. Firstly, the testbed does not account for any secondary weather effects to do with the movement of an AV or the likely position of a given sensor on an AV such as road spray, reduced visibility due to mud and dust build-up and sensor window wetting due to the dynamics of water droplets hitting and travelling across a moving vehicle. Second, the location of the testbed meant that it experienced very few instances of extreme fog or snow so statistical analysis of sensor performance under these weather conditions will not be as thorough as more common weather conditions in the area like rain. Furthermore, locating the testbed in a field means there are some distinct differences between the testbed and the environment likely to be experienced by an AV, including but not limited to the ground being grass rather than tarmac, which could affect levels of reflection and scattering from the ground. Finally, the testbed was remotely operated and this impacted the speed at which downed sensors could be recovered. This means that there are days within the dataset for which not all sensor and weather data is available.

Further data from this dataset has been made publicly available via the data portal https://saf.npl.co.uk/ (accessed on 28 August 2026). Future work on the data generated by this study will include a statistical analysis of weather parameter and sensor performance uncertainty under a given weather condition across multiple instances over the 2-year operational period.

This study set out to investigate the extent to which an outdoor testbed, with both sensor and meteorological variables measured and their uncertainties quantified, can explain and quantify the behaviour of situational awareness sensors in different types of weather with a view to use this information to explicitly determine safety limits for ODD weather parameters. From the results and discussion here, we conclude that the inescapable uncertainties in this weather sensor characterisation are significant enough to make the explicit determination of an AV remaining within its ODD solely from monitoring the COD in operations very challenging. This would require initial AV deployments operating in this manner to take a very conservative approach to the weather. Alternatively, other approaches, such as an implicit determination of the AV sensor’s current performance by the AV itself, might enable the ODD to be expanded to allow for AV operation in a wider range of weather conditions. However, caution should be taken that any such approach relying on AI algorithms ensures that the training data has sufficient coverage of scenes in different variations in weather conditions. Ideally, training datasets would be created with accompanying meteorological measurements so coverage of a wide range of weather parameter values could be guaranteed. Finally, when considering how the results from this study and others may be combined with an effort towards standardisation, a key recommendation of this paper is that all testbed operators adopt the approach taken here of measurement/parameter separation and unambiguous parameter definition. This is important for cross-comparisons of datasets.

## Figures and Tables

**Table 1:** Meteorological instrumentation deployed at the testbed. Rows shaded in grey indicate those instruments that were used to generate parameters used in this paper.

Instrument Type	Location	Model	Used For
Present weather/ visiometer	A B C	Campbell Scientific CS125	Meteorological Optical Range (MOR) in non-precipitating weather
Aerosol spectrometer	B	Grimm spectrometer (with pre-drying of aerosol)	Aerosol size distributions, optical depth (0.25–30 µm diameter)
Fog spectrometer	B	Droplet Measurement Technologies (DMT) FM120	Liquid water content, mean radius, droplet spectra (2–50 µm diameter), input to fog attenuation coefficient estimates and MOR in precipitation
Precipitation spectrometer	B	DMT Meteorological Particle Spectrometer	Investigating particle types in frozen precipitation
Disdrometer	A B C E F	Theis disdrometer	Raindrop size distribution (0.1 to 8 mm), rain fall rate, solid precipitation rate, hydrometer type, input to rain attenuation coefficient estimates and MOR in precipitation
Disdrometer	B	OTT radar disdrometer	Assess application of this non-optical device
Rain gauge	B	Tipping bucket rain gauge	Rainfall accumulation (and rate in intense rain)
Rainfall radar	-	Met Office C-band radar network	Testbed situational awareness, rainfall type
Pyranometer	A, C, H	Kipp & Zonen CMP21	Total downward short-wave flux over the range 285 to 2800 nm, Site H included a pyranometer with solar tracker for direct/diffuse calculation, combined with solar angles to specify illumination environment
Pyrgeometer	A, C, H	Kipp & Zonen CGR4	Total downward long-wave flux in the range 4.5 to 42 µm, Site H included both upward long-wave flux, contributes to thermal modelling of targets to flag possible condensation
Lux meter	A, C, H	Skye SKL510	Downward short-wave flux in human photopic waveband
Sunshine recorder	A, C, H	Kipp & Zonen CSD3	Sunshine duration recorder
Sun photometer	H	Cimel PHOTOMETER CE318-TS9	Spectral and angular radiance detail is provided at selected wavelengths across solar spectrum
Ceilometer	H	Vaisala CT25K	Provides information about cloud base and aerosol
Video camera/webcam	Various	On-site webcams	Testbed situational awareness, esp. in the event of fog or snow at the site
Wind (10 m)	G	Sonic anemometer	A single-location high-quality measurement that is representative of the site
Temperature/humidity (1.5 m)	G	PRT (temp), LiCor & Krypton	Representative temperature and humidity for the site
Grass tip temp	G	IRT	Radiative temperature of grass surface

**Table 2:** Meteorological parameters used in this paper.

Parameters	Description
Solid precipitation rate	The testbed mean solid precipitation rate (as liquid water equivalent in mm/h) provided as a factory parameter from the five disdrometers. The associated uncertainty is the standard deviation of these measurements.
Rainfall rate	The expected value of 1 min rainfall rate (in mm/h) for any disdrometer randomly located within the testbed area ACEF and derived directly from the 1 min quality-controlled disdrometer drop size distributions (DSDs) integrated across their droplet volume and theoretical terminal velocities. The derivation of these values and their uncertainties is given below.
Rainfall attenuation coefficients	Two-way attenuation coefficients (dB/km), and their uncertainties, were estimated for a number of wavebands of interest, from the disdrometer DSDs using a spherical droplet assumption and applying Mie theory, this time integrating over the size-specific extinction coefficients for each droplet size. As with rainfall rate they should be interpreted as the expected value of attenuation coefficient that would be inferred from a randomly located disdrometer. Results for 77 GHz are shown in this paper.
MOR	Meteorological Optical Range (MOR) (in m) is derived in two ways: MOR_fog_: Mean of the MOR measured by the three CS125s. Min and max values of these measurements are also provided to demonstrate the degree of horizontal homogeneity. MOR_rain_: Calculated directly from DSD-derived attenuation coefficients from disdrometers and fog spectrometer. Min and max values are calculated in line with uncertainties for rainfall rate and attenuation. (See main text for explanation)
Fog liquid water content (Site B)	Fog liquid water content (LWC) in g/m^3^ was taken as a factory parameter from the FM120 fog spectrometer.

## Data Availability

Dataset available on request from the authors.
